# Synthesis and Characterization of a New Class of Chromene-Azo Sulfonamide Hybrids as Promising Anticancer Candidates with the Exploration of Their EGFR, *h*CAII, and MMP-2 Inhibitors Based on Molecular Docking Assays

**DOI:** 10.3390/ijms242316716

**Published:** 2023-11-24

**Authors:** Fawzia F. Alblewi, Mosa H. Alsehli, Zainab M. Hritani, Areej Eskandrani, Wael H. Alsaedi, Majed O. Alawad, Ahmed A. Elhenawy, Hanaa Y. Ahmed, Mohamed S. A. El-Gaby, Tarek H. Afifi, Rawda M. Okasha

**Affiliations:** 1 Chemistry Department, College of Science, Taibah University, Medina 30002, Saudi Arabia; fbalawi@taibahu.edu.sa (F.F.A.); zozohrytany@hotmail.com (Z.M.H.); aeskandrani@taibahu.edu.sa (A.E.); wsadi@taibahu.edu.sa (W.H.A.); 2 Center of Excellence for Nanomaterials for Clean Energy Applications, King Abdulaziz City for Science and Technology (KACST), Riyadh 12354, Saudi Arabia; moalawad@kacst.edu.sa; 3 Chemistry Department, Faculty of Science, Al-Azhar University, Nasr City 11884, Egypt; elhenawy_sci@hotmail.com (A.A.E.); m_elgaby@azhar.edu.eg (M.S.A.E.-G.); 4 Chemistry Department, Faculty of Science and Art, AlBaha University, Al Bahah 65731, Saudi Arabia; 5 The Regional Center for Mycology and Biotechnology, Al-Azhar University, Nasr City 11884, Egypt; hanaayoussef@azhar.edu.eg

**Keywords:** 4*H*-chromene, sulfonamide, EGFR, MMP2, molecular docking

## Abstract

In this study, novel selective antitumor compounds were synthesized based on their fundamental pharmacophoric prerequisites associated with EGFR inhibitors. A molecular hybridization approach was employed to design and prepare a range of 4*H*-chromene-3-carboxylates **7a**–**g**, **8**, and **11a**–**e** derivatives, each incorporating a sulfonamide moiety. The structures of these hybrid molecules were verified using comprehensive analytical and spectroscopic techniques. During the assessment of the newly synthesized compounds for their anticancer properties against three tumor cell lines (HepG-2, MCF-7, and HCT-116), compounds **7f** and **7g** displayed remarkable antitumor activity against all tested cell lines, outperforming the reference drug Cisplatin in terms of efficacy. Consequently, these promising candidates were selected for further investigation of their anti-EGFR, *h*CAII, and MMP-2 potential, which exhibited remarkable effectiveness against EGFR and *MMP2* when compared to Sorafenib. Additionally, docking investigations regarding the EGFR binding site were implemented for the targeted derivatives in order to attain better comprehension with respect to the pattern in which binding mechanics occur between the investigated molecules and the active site, which illustrated a higher binding efficacy in comparison with Sorafenib.

## 1. Introduction

A group of chronic disorders known collectively as cancer is characterized by unchecked cell proliferation that is capable of infiltrating other tissues. The available therapeutic methods (surgery, radiotherapy, and chemotherapy) are invasive, and their high toxicity constrains the majority of healthy cells, which compromises the efficacy of therapy, produces a variety of side effects that have a prominent detrimental impact on general well-being, and results in significant monetary losses measured by disability-adjusted life years (DALYs) [1,2,3]. As a result, researchers have looked into new sources of anticancer chemicals in an effort to create affordable, less harmful medications. One of the various methods that have been successfully used to create and produce new and effective chemotherapeutic drugs is “molecular hybridization” [4]. The process of molecular hybridization entails joining or fusing two different pharmacophores or chemical entities to create new hybrid moieties, which are designated as “molecular scaffolds” [4,5]. Based on the bio profiles of the chosen pharmacophores or chemical moieties, the hybrid compounds are anticipated to exhibit synergistic or additive pharmacological actions [6,7]. In this regard, the substantial medicinal application scope of 4*H*-chromene heterocyclic compounds has prompted their nomination in this study as a pharmacophore in the formation of a new molecular scaffold [8,9].

**Chart 1:**
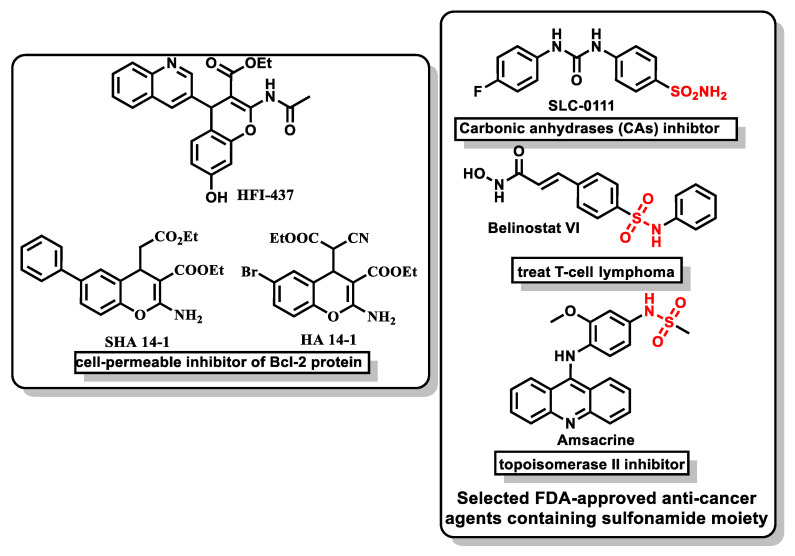
Selected potential antitumor compounds.

Heterocyclic substances are crucial to creating pharmaceuticals, as they are typically present in natural goods and biologically active substances (including chromene and its analogs). In recent years, extensive research efforts have been dedicated to synthesizing various series of chromene compounds, unveiling their exceptional potential in addressing a wide range of medical conditions and enhancing their biological effectiveness [10]. These applications include antibacterial [11,12], anticancer [13,14], anticholinesterase [15], antidepressant [16], antidiabetic [17], antiepileptic [18], antifungal [19], anti-gastric cancer [20], anti-HIV [21], antihypertensive [22], anti-inflammatory [23], antimalarial [24], antioxidant [25,26,27], antiproliferative [28], antituberculosis [29], and antivascular [30] applications. In addition, they are potential succinate dehydrogenase inhibitors [31] and tyrosinase inhibitors [32], and they have utility as raw materials in dye and pigment production [33]. The 2-amino-4*H*-chromene skeleton is one of the preferred structural motifs in a wide range of natural products, as well as pharmaceuticals with a plethora of pharmacological effects, including anticancer, antibacterial, antioxidant, and antihypertensive properties; Chart 1 [34,35,36,37]. For instance, the high-affinity insulin-controlled aminopeptidase inhibitor 2-amino-4-aryl-4*H*-chromene (HFI-437) has the potential to be implemented in the treatment of neurodegenerative illness [34]. A tubulin inhibitor known as compound MX 58151 was employed; it binds at or near the colchicine site of tubulin [35]. EPC2407 (crinobulin) is also a potential apoptosis inducer and vascular-targeting anticancer drug in the therapy of progressed solid tumors [36]. In leukemia cells, HA 14-1 and sHA 14-1 may reduce drug resistance and work in concert with a number of cancer treatments [37].

The integration of molecular hybridization in the antitumor therapeutics drug design and development domain has been a relatively recent conception and a compelling instrument in the construction of physiologically active drugs [38,39,40,41]. The manufacture of these hybridized molecules maintains their primary properties (with the exception of superior molecular masses and lipophilicity, violating Lipinski’s and Veber’s rules) through the focal fusion of biologically active/pharmacophoric molecules into a unique architecture with the amplification of their applicable characteristics exceeding the summation of the precursors’ potencies [38,39,40,41,42,43,44,45,46]. These hybridized antitumor agents exhibit eminent benefits in comparison with their traditional analogs due to their inclination to synchronously interact with diverse biological targets (enhanced affinity), which diminishes drug–drug dynamics and subsequently diminishes prospective additive adverse effects and their elicitation of the resistance induced towards the parent molecules (enhanced efficacy and safety) [38,39,40,41,42,43,44,45,46].

Similarly, the distinctive and versatile sulfur-containing drugs enable sulfonamides or sulfonyls to occupy a significant stance in the medicinal world [47,48,49,50,51], facilitating their integration as an additional pharmacophore in constructing a synthetically enhanced molecular scaffold. Since the discovery of the first successful antimicrobial sulfonamide drug Prontosil rubrum, around 100 FDA-established potential sulfonamide drugs are available in the market [52] that have a huge range of therapeutic antibiotic and anticancer applications [53,54,55,56,57,58,59,60,61,62,63]. Evidently, the FDA has granted clearance to several sulfonamide molecules in tumor treatment. For example, SLC-0111 is currently undergoing clinical investigations as a standalone drug for solid tumors and exhibits significant selectivity towards the CA IX isoform [54]. Furthermore, as a histone deacetylase (HDAC) inhibitor, belinostat (VI) is permitted for the therapy of T-cell lymphoma [55]. Amsacrine (VII), a topoisomerase II inhibitor, is licensed to remedy tumorous lymphomas and acute leukemias by integrating into the DNA of cancer cells; Chart 1 [56].

**Figure 1:**
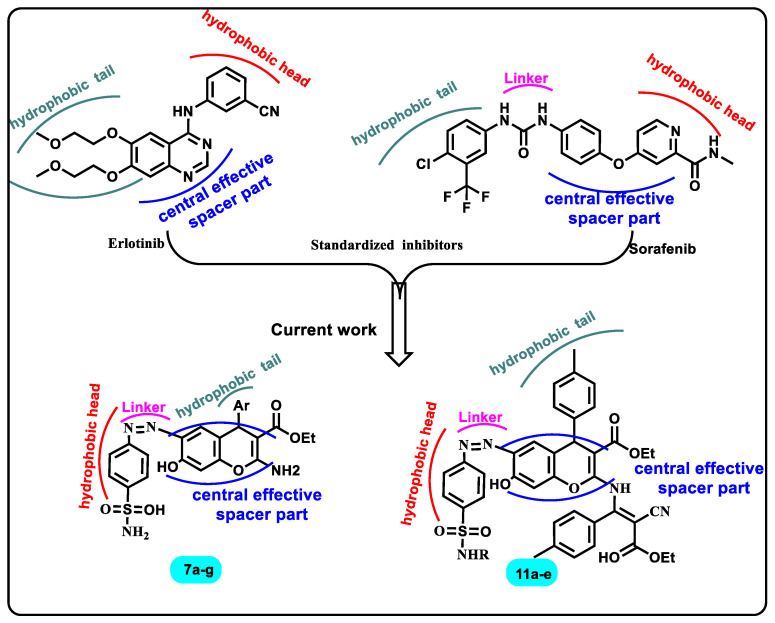
Rational design and structural modification of chromene-azo sulfonamide hybrids.

These cancer therapeutic drug nominations (both the heterocyclic chromenes and the sulfur-integrating derivatives) are enabled to inhibit the overexpression of *EGFR* (epidermal growth factor receptor), which triggers the emergence of numerous tumor subtypes (notably colon, breast, and ovarian). This receptor is responsible for the regulation of cell proliferation and/or apoptosis through the diverse signal transduction pathways. It was discovered to contribute to angiogenesis, which enhances the proliferation, infiltration, and metastasis of malignant cells [64]. Subsequently, one of the most efficacious FDA-affirmed drugs in cancer therapeutics is an *EGFR* inhibitor, referred to as Erlotinib [64]; Figure 1. According to the aforementioned information, the inhibition of *EGFR* offers a highly viable cancer therapeutic strategy [65]. We can observe from Figure 1 that the conventional pharmacophoric characters of *EGFR* inhibitors require the presence of a hydrophobic head, a spacer at the heteroaromatic ring, and a hydrophobic tail.

The pairing of sulfonamides with various heterocycles, including thymol, cumene, indazole, pyrazole, pyridazines, triazoles, coumarin, and chromene, has demonstrated significant success in the pharmaceutical field, and ongoing advancements are currently undergoing clinical trials [64,65,66]. Based on this, and according to the aforementioned facts, which have encouraged our research interest in 4*H*-chromene derivatives and sulfonamides as pharmacophores, this study intends to establish a chromene–sulfonamide hybrid via the molecular hybridization methodology in order to construct EGFR inhibitors and assist in the discovery of new therapeutic innovations [67].

### Rationale and Work Design

In light of the aforementioned data and reasoning, 4*H*-chromene-azo sulfonamides were composed and configured as prospective anticancer candidates for the inhibition of EGFR receptors. Concisely, the key pharmacophoric characteristics of EGFR inhibitors are depicted in Figure 1 as follows: the hydrophobic head must be bound to the hydrophobic area for the EGFR binding pocket, the flat heteroaromatic ring must be bound to the adenine binding pocket, and the hydrophobic tail must be bound to the hydrophobic region. Therefore, the newly designed compounds are instituted based on the introduction of the azo-sulfonamide scaffold as the hydrophobic tail, which binds to hydrophobic region II of EGFR. Additionally, the central 4*H*-chromene spacer moiety is designed to bind to the adenine binding pocket of EGFR. Lastly, different head rings were incorporated to inhibit the hydrophobic region of EGFR. Consequently, regardless of the absence of some of the conventional pharmacophoric properties in these novel prospects, this novel formulation intends to target the EGFR receptor. Several of the novel derivatives’ distinctive designs might provide enough flexibility to adhere to the receptors, which proves valuable. Furthermore, sulfonamides are designated to be in agreement with the general pharmacophoric requirements for hCA II inhibition: an aromatic sulfonamide moiety is used as a base unit for the synthesis of the target compounds (the binding coordination of the Zn atom and the amino acids is in the active site pocket). The 4*H*-chromene spacer ring is attached to the aromatic scaffold and used as a hydrophilic linker to incorporate several substitution patterns planned to increase the hydrophobic interactions within the active site cavity; Figure 1.

## 2. Results and Discussion

### 2.1. Chemistry

**Scheme 1:**
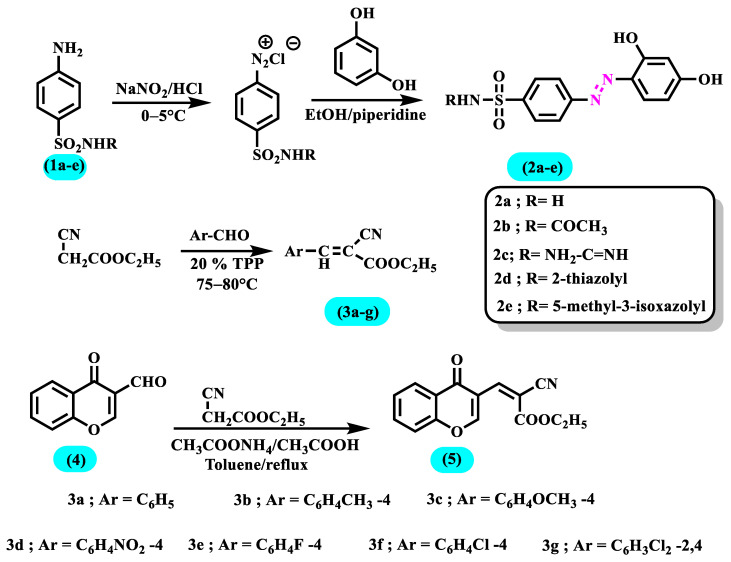
The key intermediates 4-((2,4-dihydroxyphenyl)diazenyl) benzene sulfonamides **2a**–**e**, α-Cyanoacrylates **3a**–**g**, and 2-cyano-3-(4-oxo-4*H*-chromen-3-yl) acrylate **5**.

Scheme 1 portrays the synthesis of three key intermediates, **2a**–**e**, **3a**–**g**, and **5**, which are implemented in the synthetic protocol for this report. The key intermediates 4-((2, 4-dihydroxyphenyl)diazenyl)benzene sulfonamides **2a**–**e** were constructed through the coupling of diazotized 4-aminobenzene sulfonamides **1** with resorcinol in the presence of alkali [68,69,70]. The α-cyanoacrylates **3a**–**g** were achieved in good yields, using the Knoevenagel condensation of aromatic aldehydes with ethyl cyanoacetate in the presence of 20 mol % of triphenylphosphine (TPP) at 75–80 °C [71]. Moreover, 2-cyano-3-(4-oxo-4*H*-chromen-3-yl)acrylate **5** was attained through the reaction of 3-formylchromone **4** with ethyl cyanoacetate in refluxing toluene in the occurrence of ammonium acetate and glacial acetic acid with the aid of a Dean–Stark trap; Scheme 1 [72].

In continuation with our studies [68,69,70] on the reactivity of 4-((2,4-dihydroxyphenyl) diazenyl)benzene sulfonamides **2**, the existing work assessed their performance in response with α-cyanoacrylates **3** as electrophiles. The current investigation incorporates different substituents on the chromene moieties in order to optimize the biological performance of the target drug candidates. In the literature, there is a comparative analysis between molecules integrating nitrile functional moieties versus alkoxy carbonyl motifs in cancer therapeutics research [35,36,70]. Both possess promising antitumor activities; however, the anticancer performance varies in accordance with the molecular structure and the type of cancer cell line. In our current research, the alkoxy carbonyls have attained higher antitumor functionalities in an evaluation against the same cancer cell lines and in comparison with our previous work [70]. The reaction of compound **2a** with the appropriate α-cyanoacrylates **3** yielded the corresponding 4*H*-chromene-3-carboxylates **7a**–**g** rather than the expected compounds **6**; Scheme S1. The reactions were conducted in a 1:1 molar ratio in refluxing ethanol in the existence of a catalytic quantity of piperidine. The infrared spectra of derivatives **7a**–**g** revealed the shortage of nitrile absorption bands and the presence of the NH_2_, C=O, and N=N distinctive absorption bands, Figures S2–S4. The IR spectrum of molecule **7a** taken as a representative example of the series showed characteristic absorption bands at 3358, 1673, 1514, and 1071 cm^−1^ stretching vibrations of NH_2_, C=O ester, N=N, and SO_2_ functional groups, respectively. The structure of compounds **7a**–**g** was supported by the ^1^HNMR spectra, which showed the presence of the expected proton signal in accordance with the suggested structure, Figures S7–S11. The representative ^1^HNMR spectrum of compound **7b** (DMSO-d6) revealed a triplet at δ 1.16, a quartet at δ 4.01 (assigned to the ethyl group), a singlet at δ 2.20 (ascribed to the methyl protons), and two singlets at δ 7.52 and 7.68 (ascribed to the NH_2_ protons). The signal that appeared at δ 5.07 (assigned to one proton) is a result of the chiral C–H of the pyran ring in addition to the downfield singlet at δ 12.04 (ascribed to the OH proton). The ^13^C-NMR spectrum of molecule **7b** revealed signals at δ 20.98 (Ar-CH_3_), 34.24 (pyran-CH), and 77.48 (pyran-C3). The signals that appeared at δ 14.81 and 59.23 can be assigned to the methyl carbon and methylene, respectively. The most downfield signal appeared at δ 168.46, which can be appointed to the carbonyl group.

The formation of 4*H*-chromene-3-carboxylates **7a**–**g** is explicated through the reaction pathway, demonstrated in Scheme S2. The synthesis of **7** is postulated to persist through the Michael addition pathway of the carbanion derived from **2**, which primarily contributes to the energized double bond of derivative **3** to develop the analogous Michael adduct (A). Subsequently, the oxygen atoms’ nucleophilic assault on the cyano group’s carbon atom results in an O-heterocyclization product that is stabilized as the 2-amino-4*H*-chromene-3-carboxylate **7** tautomer; Scheme S2. Additional substantiation for the contemplated configuration of **7** was attained through the independent formation of molecule **7** via the ternary condensation reaction of molecule **2**, aromatic aldehyde, and ethyl cyanoacetate (1:1:1 molar ratio) in refluxing ethanol in the occurrence of a catalytic quantity of piperidine to yield an output equivalent in all respects (m.p., TLC, and spectra) to the resultant products acquired through the preceding reaction of **2** with **3**. Analogously, molecule **2a**, Figures S5 and S6, interacted with 2-cyano-3-(4-oxo-4*H*-chromen-3-yl)acrylate **5** and afforded the ethyl 2′-amino-7′-hydroxy-4-oxo-6′-((4-sulfamoylphenyl)diazenyl)-4*H*,4′*H*-[3,4′-bichromene]-3′-carboxylate **8**. The architecture of molecule **8** was designated according to its elemental analysis and spectral data. The IR spectrum of derivative **8** demonstrated the characteristic absorption bands for NH_2_, C=O ester, N=N, and SO_2_ at 3300, 3114, 1746, 1613, 1502, and 1129 cm^−1^, respectively. The ^13^C NMR data revealed the appearance of signals at δ 19.67 (CH_3_), 34.59 (pyran-C4), 63.89 (CH_2_), 77.35 (pyran-C3), and 169.25 (pyran-C2). The most downfield signal appeared at δ 173.25 and 180.65, which can be assigned to the two carbonyl carbons of ester and chromenone, respectively; Scheme S1.

Furthermore, this manuscript encompasses the synthesis of unanticipated new classes of molecules with an alkoxy carbonyl substitution; Scheme S3. This investigation was expanded to analyze the behavior of **2a**–**e** with two-fold α-cyanoacrylate **3b** with the aim of synthesizing novel condensed chromenes. Thus, the treatment of α-cyanoacrylate **3b** with compounds **2a**–**e** (1:2 molar ratio) in refluxing ethanol in the incidence of piperidine afforded the 4*H*-chromene-3-carboxylates **11a**–**e**. The structures of chromenopyridines **9** and **10** were ruled out on the premise of the analytical and spectral evidence.

The infrared spectra of molecules **11a**–**e** indicated characteristic absorption bands at 2217–2223 cm^−1^ for the CN group. Moreover, the infrared spectra revealed absorption bands for two carbonyl groups at 1721–1723 and 1598–1682 cm^−1^ alongside the absorption for the N=N group at 1457–1497 cm^−1^. The ^1^H-NMR spectra of compounds **11a**–**e** demonstrated a singlet signal at δ 5.01–5.08 for the pyran H-4, Figures S12–S14. The ^1^H-NMR spectrum of molecule **11a** revealed, beside the expected signals, two triplets at δ 1.17 and 1.30 and two quartets at δ 4.02 and 4.31, assigned to the two ethyl groups. The mass spectrum of compound **11a** disclosed a molecular ion peak at *m*/*z* = 721 (M+; 35.49%), which is in accordance with the assigned molecular formula C_38_H_35_N_5_O_8_S. Furthermore, the mass spectrum of **11e** presented an ion peak at *m*/*z* = 802 (M+; 39.63%), which is in correspondence with the molecular formula C_42_H_38_N_6_O_9_S. A proposed methodology in the formation of 4*H*-chromene-3-carboxylates **11** is illustrated in Scheme S3. The approach undertaken for the development of **11** from the interaction of α-cyanoacrylate **3b** and compound **2** is presumed to progress via the preliminary composition of 4*H*-chromene-3-carboxylate **7**, followed by the Michael-type addition conjugation of the amino group of **7** to the energized double bond in **3** to construct the non-isolable intermediate C, which is oxidized in accordance with specific reaction conditions to form 4*H*-chromene-3-carboxylate **11**; Scheme S3.

### 2.2. Biological Screening

#### 2.2.1. Cytotoxic Screening

**Figure 2:**
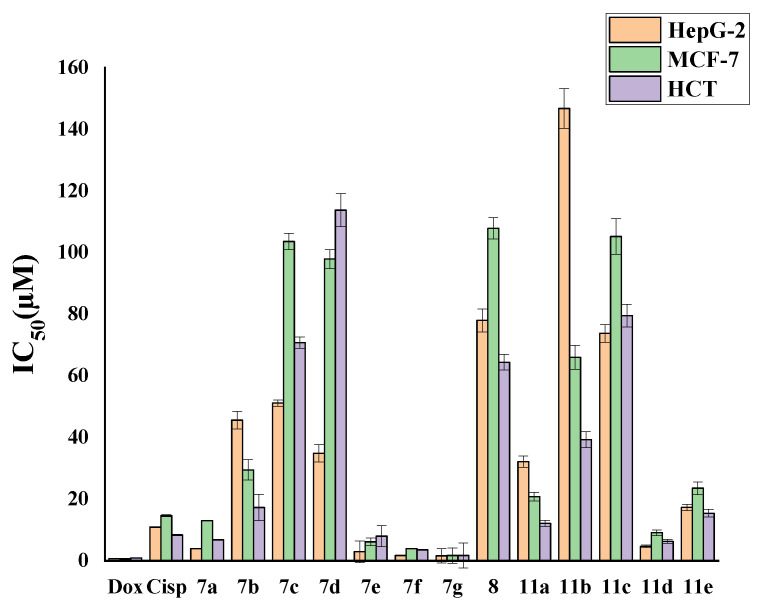
In vitro cytotoxic activity of the target compounds **7a**–**g**, **8**, and **11a**–**e** against Dox. (Doxorubicin), and Cisp. (Cisplatin) against three human carcinoma cell lines.

Three human tumorous cell lines—human colon carcinoma (HCT-116), human liver carcinoma (HepG-2), and human breast adenocarcinoma (MCF-7)—were subjected to cytotoxic behavior, employing the MTT analysis [73]. Moreover, a normotoxicity assessment was employed against normal Vero cell line (African Green Monkey Kidney cells) for selected active compounds to determine their therapeutic safety. The Cisplatin and Doxorubicin were utilized as a positive control, exhibiting elevated cytotoxic activities. The inhibitory performance of derivatives **1a**–**e**, **2a**–**e** [70], **7a**–**g**, **8**, and **11a**–**e**, and its derived relation to the cell proliferation of these three cancers, are described in Table 1 and Figure 2. The sulfa molecules class **1a**–**e** illustrated an inferior potent performance under consideration as cell growth inhibitors. Characteristically [70], the inhibitory activity was enhanced via incorporating the azo moieties in compounds **2a**–**b**. Compounds **11a** and **11c**–**e** displayed particularly poor normotoxic behavior in comparison with the cytotoxicity on the tumor cell lines, which establishes their beneficial prospect as anticancer agents.

Furthermore, the cytotoxic and potent properties of these molecules were enhanced through the inclusion of chromene ester motifs, which were compared to reference drugs. The results revealed that the halogenated aryl functional groups displayed superior activity when compared to their 4-aryl counterparts and the unsubstituted rings among the azo/chromene ester-based sulfonamides. The dichloro aryl substituents **7g**, bound to the azo molecule of the sulfanilamide scaffold, revealed the greatest potency and antiproliferative impact, proceeded by the 4-chloro (**7f**) and 4-fluro (**7e**) derivatives of the chromene compound as compared with the unsubstituted 4-aryl (**7a**). On the other hand, compound **11d**, where 4-methyl chromene ester is linked to sulfathiazole, showed improved inhibitory activity compared to the other four sulfa derivatives **11a**–**c** and **11e**.

#### 2.2.2. Structure–Activity Relationship (SAR) Study

**Chart 2:**
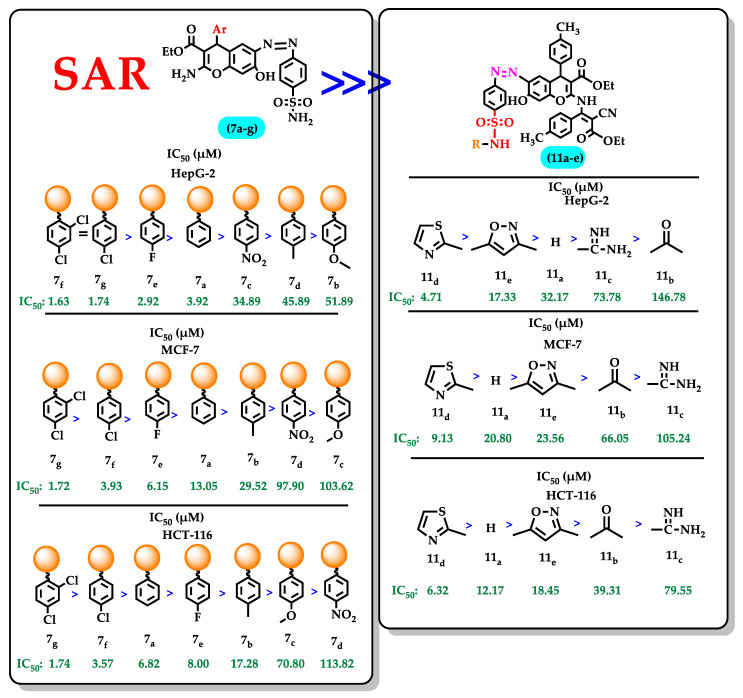
Structure–activity relationship of the newly synthesized **7a**–**g** and **11a**–**e** compounds.

The SAR study deals with the phenyl group at 4*H*-chromene **7a**–**g**. The order of potency was dependent on the type of substitution on the phenyl group, and their performances against HepG-2 cancer cells were diminished in the sequence of 2,4-Cl ≈ 4-Cl > 4-F > phenyl > NO_2_ > 4-CH_3_ > 4-OCH_3_ with IC_50_ = 1.63–51.89 μM. Further investigations regarding the potency of **7a**–**g** against the MCF-7 cancer cells revealed that the anti-proliferative activities were decreased in the order of 2,4-Cl > 4-Cl > 4-F > phenyl > 4-CH_3_ > NO_2_ > OCH_3_ with IC_50_ = 1.72–103.62 μM. On the other hand, molecules **7f** and **7g** offered good potency against the HCT-116 cell lines. This result indicates that inserting lipophilic electron-withdrawing substituents of moderate sizes, such as halogen, is more valuable for the activity than an electron-donating substituent, such as methyl or methoxy groups. Besides increasing with donating groups, **7g** may be advantageous. Furthermore, compounds **8** and **11a**–**e** had weak activity against all cell lines, indicating that when blocking SO_2_NH_2_, a decrease in activity was detected; Chart 2.

#### 2.2.3. Anti-EGFR Activity

**Figure 3:**
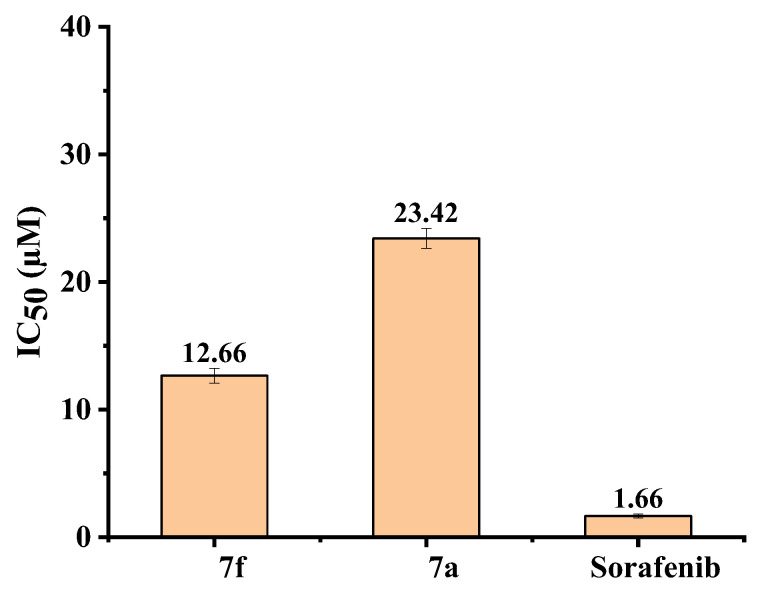
IC_50_ value of compounds (**7f** and **7a**) on EGFR compared to Sorafenib.

The epidermal growth factor receptor (EGFR) is a transmembrane glycoprotein that is one of four tyrosine kinase receptors in the erbB family. The physiological role of the epidermal growth factor receptor (EGFR) is to control the growth and homeostasis of epithelial tissues; thus, its overexpression fuels tumorigenesis. The amplification and point mutations at the genomic locus are the main causes of activation of the EGFR in cancer. The resistance of tumor cells to chemotherapy and radiation therapy is also influenced by EGFR activation. Over the past 20 years, much work has been put into creating anticancer drugs/inhibitors that can thwart EGFR function, and they can sometimes result in tumor regression while also stopping tumor development [74,75]. The current study revealed the ability of two compounds (**7a** and **7f**) to inhibit EGFR activity; Figure 3. The IC_50_ values of the two compounds (**7a** and **7f**) showed the highest percentage inhibition towards EGFR in comparison with the reference drug utilized in this experiment, Sorafenib; Figure S15. Meanwhile, from the results in Figure 3, it was observed that the IC_50_ value of molecule (**7f**) was approximately 12.66 µM, which is higher than the IC_50_ value of molecule (**7a**), which was approximately 23.42 µM. In comparison with the reference drug Sorafenib, the IC_50_ value is approximately 1.66 µM. According to our results, derivative (**7f**) exhibited superior potency in comparison with derivative (**7a**) but inferior potency in comparison with Sorafenib.

#### 2.2.4. MMP-2 Inhibition

**Figure 4:**
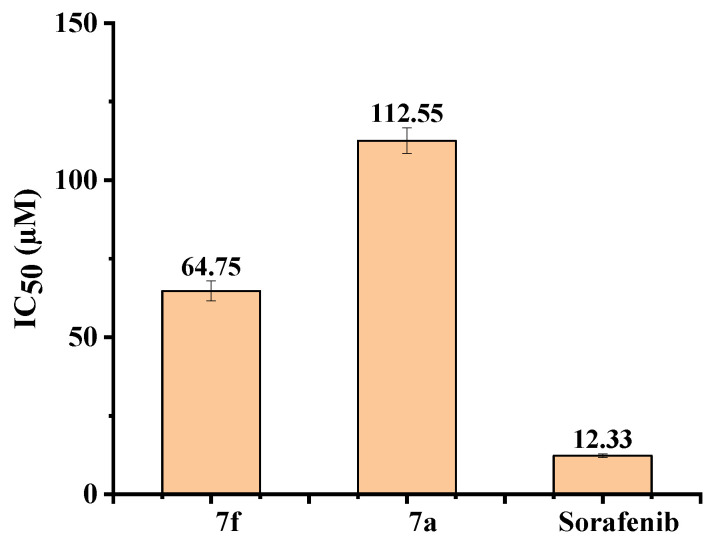
The inhibitory activity by IC_50_ of compounds (**7f**, **7a**, and Sorafenib) on MMP-2.

Cancer is a group of disorders characterized by abnormal cells that proliferate uncontrollably and have the potential to infiltrate other parts of the body. Uncontrolled cell proliferation can create a tissue mass known as a primary tumor [76]. Metastasis is the process through which cancer cells destroy the tissues around them, get past biological barriers, settle down, and multiply in a different body area to create a new tumor. Inhibiting metastasis is critical for successful anticancer therapy [77]. Therefore, in our study, derivatives **7f** and **7a** demonstrated inhibitory activity in cell detachment by inhibiting the expression of the MMP-2 enzyme, which is deemed a critical component in cancer cell survival and expansion as it is involved in all stages of carcinogenesis. Following treatment, the inhibitory activity was evaluated with two-fold serial dilutions of active compounds. The results elucidated that the potency of the anti-MMP-2 performance approached approximately 80.93% in the case of molecule **7f** and 73.89% for molecule **7a** at the first concentration as compared with Sorafenib, which reached about 95.37% at the same concentration; Figure S16. The data illustrated in Figure 4 revealed that the lower IC_50_ value of compound **7f** was approximately 64.75 µM, followed by compound **7a** at approximately 112.55 µM for MMP-2. In contrast, the IC_50_ value of Sorafenib (reference drug) is about 12.33 µM.

#### 2.2.5. Carbonic Anhydrase CAII inhibition

**Figure 5:**
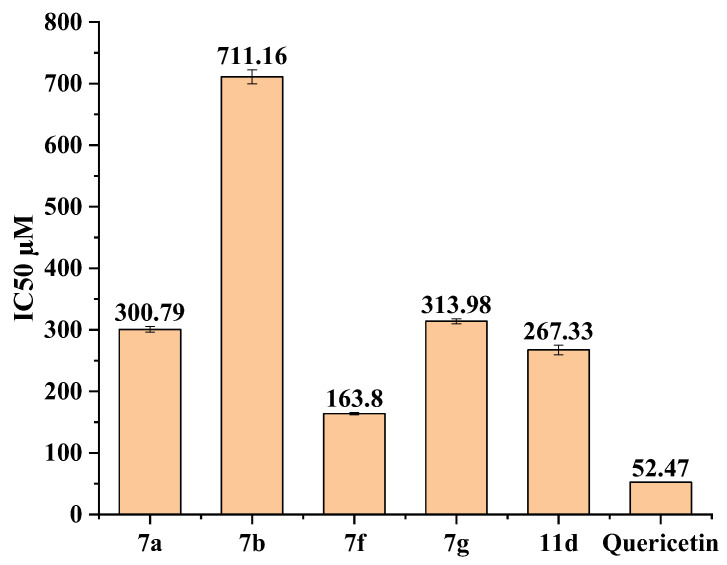
The inhibitory activity by IC_50_ of **7a**, **7b**, **7f**, **7g**, and **11d** against hCA II compared to Quercetin.

The most active synthesized compounds **7a**, **7b**, **7f**, **7g**, and **11d** were scrutinized against the cytosolic hCA II by reduction in esterase activity; results are presented in Figure 5. The cytosolic hCA II was moderately inhibited by all the tested compounds, with IC_50_ ranging from 163.8 to 711.16 µM in comparison with the Quercetin control (52.47 µM). The most active compounds were the 4*H*-chromene derivatives **7f** and **11d** (163.8 and 267.33 µM, respectively), Figure 5 and Figure S17.

Moreover, the lowest behavior was observed from the parent molecule, bearing the tolyl fragment **7b** (711.16 µM). A relatively poor affinity for the remaining investigated derivatives (**7a** and **7g**) was observed against hCA II, with their inhibitory activity spanning between 300.79 and 313.98 µM, respectively.

### 2.3. Molecular Docking Analysis

The docking analysis was performed to explicate the potency of these desirable molecules in vitro against the EGFR, MMP-2, and hCAII proteins, respectively, through their potential interaction mechanisms with their crystal frameworks (PDB: 4HJO [77], 1HOV [78], 3M04 [79]). The docking investigation procedures were implemented using Glide’s module^®^ [80,81,82,83,84]. The preliminary inhibitors (Erlotinib, hydroxamic acid, and sulfonamide) were redocked into the examined crystal frameworks to verify the docking methodology. Furthermore, the efficacious performance of the targeted molecules was authenticated via the low values of RMSD (1.02 and 1.9 Å for EGFR, and 1.02 and 1.62 A for MMP-2 and hCA II, respectively), which were acquired through the root mean square deviation between the native and redocked poses of the co-crystallized inhibitor. The binding free energies Δ*G* are listed in Table 2 and Supplementary Materials Table S1.

The initial inhibitors have been adequately installed into their binding sites in order to attain their crystal configurations. The lowest score poses and RMSD revealed increased stability in the binding pocket. The data were utilized to rank the docked poses and to select the most capable docked conformation of each compound. The hetero ring for Erlotinib and benzimidazole centered on the EGFR adenine pocket and interacted with ASN54, ASP89, Met 769, Gly772, Asp831, Lys721, and Leu694, while the hydroxamic acids are capped with Tyr74, Ala74, and Leu137, and 143 in MMP-2. Sulfonamide is chelated with vital amino acids for hCAII, including Asn67, His119, Thr199, Asn62, HIS94, His96, and ZN. The molecule docked prolifically into active sites in a similar mechanism as that of the original inhibitors.

#### 2.3.1. In Case of EGFR Protein

**Figure 6:**
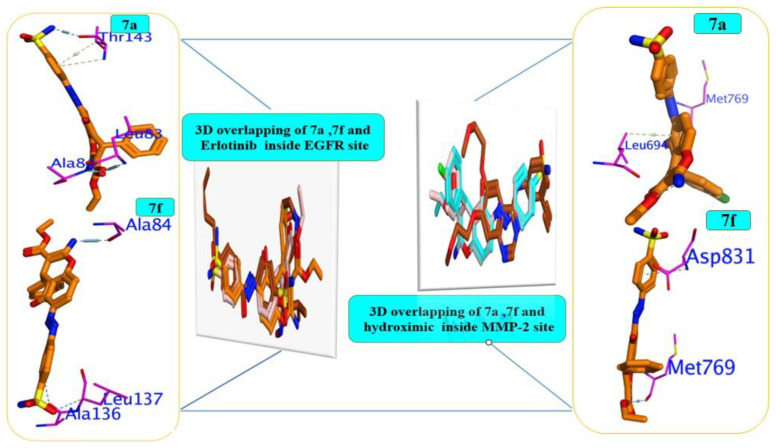
Three-dimensional docking poses of compounds **7a** and **7f** and their overlay with reference inhibitors inside active sites.

All compounds **2a**–**e**, **7a**–**g**, **8**, and **11a**–**e** demonstrated higher binding affinities (Δ*G* = −6.4 to −8.6 Kcal/mol) than Erlotinib (Δ*G* = −5.3 to Kcal/mol), which substantiated their promising potencies. The interactions between the thirteen active compounds **2a**–**e**, **7a**–**g**, **8**, and **11a**–**e** and the active site residues were mainly polar bonds, hydrogen bonding, and π−π and π−H interactions, which contributed to a strong alignment with the enzyme backbone; Figure S1. The active molecules **7a** and **7f** were attached deeply into the binding pockets, interacting with the vital hydrophobic Met769 residue in the same manner of Erlotinib. Derivative **7f** had binding energies equivalent to −8.6 Kcal/mol, conforming in the binding pocket by interacting with the hydrophobic binding pocket Met769, which led to a decrease in activity against urease (Figure 6). Compound **7a** was docked in the EGFR binding pocket via interaction with Met769 and Leu694. It is inferred that the formation of strong interactions with important residues can pinpoint the EGFR binding pocket. Lastly, according to the 3D-molecular docking, the more active **7a** and **7f** prefer a parallel orientation between the central chromene ring and the important hydrophobic Met769.

#### 2.3.2. In MMP-2 Case

The investigated diazochromenes **2a**–**e**, **7a**–**g**, **8**, and **11a**–**e** displayed lower binding affinities (Δ*G* = −7.71 to −9.98 Kcal/mol) than hydroximic acid (Δ*G* = −10.0 Kcal/mol), which substantiated their lower potencies for most active compounds than the reference Erlotinib (Table 2). The interactions between the investigated compounds and the active site residues contributed to a strong alignment with the enzyme backbone; Figure 6. The active molecules **7a** and **7f** were attached deeply into the binding pockets, interacting with the vital hydrophobic Ala84 and Leu137 residues in the same manner as that of reference inhibitor. The molecular docking showed that the more active **7a** and **7f** prefer a perpendicular orientation between the phenyl ring and important Ala84.

#### 2.3.3. In hCAII Case

Unfortunately, derivatives **7a**, **7b**, **7f**, and **11d** demonstrated low potency in comparison with Sulfonamide (Table 2 and Supplementary Materials Figure S18). In order to explicate the lower activity for these molecules, molecular docking was performed on the hCAII active site [85]. They had binding energies equivalent to −6.13 to −9.15 Kcal/mol for sulfonamide derivatives **7a**–**g**, **8**, and **11a**–**e**, which are unable to install in the binding pocket due to their inability to bind with the Zn metal, which chelates with the important amino acids Thr199, His119, and Hid 96. Thus, the synthesized derivatives exhibit moderate potency.

## 3. Methods and Materials

### 3.1. Material and Methods

Solvents and reagents, as commercial grade, were obtained from Sigma-Aldrich (St. Louis, MO, USA). Melting points were measured with a Stuart Scientific apparatus and are uncorrected. The IR spectra were measured, employing a Jasco FT/IR 460 plus spectrophotometer (Jasco, Tokyo, Japan). The ^1^H-NMR and ^13^C-NMR spectra were recorded, utilizing a Bruker AV 500 MHz spectrometer (Bruker, Billerica, MA, USA). The ^13^C-NMR spectra were obtained, where the signals of CH and CH_3_ carbon atoms appear normal (up) and the signals of carbon atoms in CH_2_ environments appear negative (down). Chemical shifts (δ) are expressed in parts per million (ppm), while the MS spectra were measured using a Shimadzu GC/MS-QP5050A spectrometer (Shimadzu, Kyoto, Japan). Elemental analyses were carried out at the Regional Centre for Mycology & Biotechnology, Al-Azhar University, Cairo, Egypt, and the results were within ±0.25%. Microwave synthesis was performed using a mono-mode Milestone Sr1 device. All the reactions were monitored using thin layer chromatography (TLC) on silica gel precoated F254 plates (Merck, Billerica, MA, USA).

### 3.2. Chemistry

#### 3.2.1. General Procedure for the Synthesis of 4-((2-Amino-4-aryl-7-hydroxy-4-aryl-4*H*-chromen-6-yl)diazinyl) Benzenesulfonamides **7a**–**g**

**Method 1:** To a mixture of compound **2a** (0.01 mol) and α-cyanoacrylates **3** (0.01 mol) in ethanol (15 mL), a few drops of piperidine were added. The reaction mixture was refluxed for 1 h. A crystalline solid was obtained on cooling. It was recrystallized from an appropriate solvent.

**Method 2:** To a mixture of compound **2a** (0.01 mol), ethyl cyanoacetate (0.01 mol), and aromatic aldehyde (0.01 mol) in ethanol (15 mL), a few drops of piperidine were added. The reaction mixture was refluxed for 1 h and poured into ice water. The solid product was collected, washed with water, and recrystallized from an appropriate solvent.

#### 3.2.2. Ethyl 2-Amino-7′-hydroxy-4-oxo-6′-((4-sulfamoylphenyl)diazenyl)-4*H*,4′*H*-[3,4′-bichromene)-3′-carboxylate 8

To a mixture of compound **2a** (0.01 mol) and 2-cyano-3-(4-oxo-4H-chromen-3-yl) acrylate 5 (0.01 mol) in ethanol (15 mL), a few drops of triethylamine were added. The reaction mixture was refluxed for 0.5 h. The solid product, so formed, was collected by filtration and recrystallized from ethanol to yield 8.

#### 3.2.3. General Procedure for the Synthesis of Ethyl 2-((2-Cyano-3-ethoxy-3-oxo-1-(p-tolyl)prop-1-en-1-yl)amino)-7-hydroxy-6-((4-sulfamoylphenyl)diazenyl)4-(p-tolyl)-4*H*-chromene-3-carboxylates **11a**–**e**

A solution of α-Cyanoacrylates **3b** (0.02 mol) in ethanol (15 mL), compound **2** (0.01 mol), and a few drops of piperidine was refluxed for 1 h and cooled. The precipitate was filtrated off and crystallized from the appropriate solvent.

The IR, NMR, MS, and elemental analysis are provided in the Supplementary Material file.

### 3.3. Biological Screening

#### 3.3.1. Cytotoxicity Evaluation Using Viability Assay

The cytotoxic activity was appraised using the 3-(4,5-dimethylthiazol-2-yl)-2,5-diphenyl-tetrazolium bromide (MTT) colorimetric assay as reported previously.

#### 3.3.2. EGFR Inhibition Activities

Target compounds were measured using the EGFR kinase assay kit (Cat. #40321, BPS bioscience, San Diego, USA). The detection kit is a luminescence kinase detection approach that detects the amount of ADP produced by the kinase reaction. After ADP is converted into ATP, ATP can be used as the substrate of the luciferase-catalyzed reaction to generate an optical signal, which is positively correlated with kinase activity. In vitro EGFR inhibitory activity of most active compounds was evaluated via Enzyme-Linked immunoabsorbent assay (ELISA) using an EGFR kinase kit (Cat. No. 40321; BPS Bioscience, San Diego, CA, USA). Briefly, solutions of kinase/peptide and ATP (25 μL) per well were prepared before use. The inhibitory solution was added at 5 μL to each well. Then, 20 μL of 1× kinase assay buffer and 20 μL of diluted EGFR enzyme were added. The plate was incubated at 30 °C for 40 min. After that, 50 μL of Kinase-Glo Max reagent was added to each well. The plate was incubated at room temperature for 15 min. The absorbance was recorded at 412 nm using a microplate ELISA reader (SunRise TECAN, Redwood City, CA, USA). Each experiment was repeated three times, and IC_50_, represented as mean ± S.E., was calculated using Graph Pad Prism 5 [86].

#### 3.3.3. MMP-2 Inhibition

In vitro MMP-2 inhibitory activity of most active compounds was evaluated via Enzyme-Linked immunoabsorbent assay (ELISA) using an MMP-2 Inhibitor Screening Assay Kit (Colorimetric) ab139446 (Abcam, Cambridge, UK) with Sorafenib as a positive control. For one well of the 96-well plate, the suggested volume of enzyme solution was 40 µL, with 10 µL of test compounds. The following control wells were simultaneously established, as deemed necessary: The vehicle control contains MMP enzyme and vehicle used in delivering test compound (DMSO, concentration not to exceed 1%). The total volume of all controls is 50 µL. The plate was pre-incubated for 10 min at an assay temperature of 37 °C. The MMP substrate solution (Ac-PLG-[2-mercapto-4-methyl-pentanoyl]-LG-OC2H5) was added to each well (50 µL). The reagents were mixed completely by shaking the plate gently for 30 sec, and were then incubated for 60 min. After that, 50 µL of stop solution was added to each well, and the absorbance was measured at 412 nm using a microplate ELISA reader (SunRise TECAN, USA). Each experiment was repeated three times, and IC_50_, represented as mean ± S.E, was calculated using Graph Pad Prism 5.

#### 3.3.4. CA-II Inhibition Assay

The CA-II inhibition assay for the analyzed molecules was performed based on the measurement of the reduction in esterase activity on the substrate p-nitrophenyl acetate (4-NPA). Briefly, the total mixture volume was 200 µL in a well containing 140 µL (20 mM HEPES) Tris buffer (pH 7.4), 20 µL of the enzyme (from bovine, Sigma–Aldrich, C2624, St. Louis, MO, USA), and 20 µL (0.5 mg/mL in DMSO) of the investigated derivatives, which were mixed and incubated at 25 °C for 15 min. After incubation, 20 µL of the substrate (4-nitrophenyl acetate, Sigma–Aldrich, N-8130; 0.7 mM in MeOH) was added. The reaction was run under the same conditions for 60 min, and a final read was taken at 405 nm. Quercetin was used as the reference drug (positive control). Subsequently, the hydrolysis of the substrate was evaluated at 405 nm, employing a microplate ELISA Reader. The results, attained after the reactions were completed in triplicate, were quantified by the equation given below. The percent inhibition for each sample was calculated as Inhibition (%) = [(C − T)/C] × 100, where C (i.e., control) = total enzyme activity without inhibitor and T (i.e., test sample) = activity in the presence of test compound. The inhibitory performance was expressed as IC_50_ values (the concentration at which 50% of the enzyme activity is inhibited), which were calculated utilizing Microsoft Excel 2011 from a dose–response curve acquired though the usage of ten tested concentrations (ranging from 0.5 to 1000 µg/mL) of the inhibitor and executed in triplicate.

### 3.4. Docking Analysis

GOLD was implemented to conduct a docking investigation for the target molecules (version 5.2) alongside its employment in the discovery studio [87]. After attaining the crystallized EGFR architecture, the H_2_O and inhibitors were removed, and the H atoms were added. The examined ligands were redocked into the vacant active site after the standard inhibitor was removed from it. The ChemPLP scoring function was generated to measure the binding affinity, and the charges were assigned with the CHARMM force field. The structure with the lowest RMSD score was utilized to generate different ligand poses. To calculate the binding affinity, the ChemPLP scoring function was integrated, and the CHARMM force field was used to assign charges.

## 4. Conclusions

Recently, the concept of molecular hybridization has been recognized as an effective approach in promoting drug discovery. A molecular hybridization methodology was integrated into the design and development of a number of novel 4*H*-chromene-3-carboxylate derivatives (**7a**–**g**, **8**, and **11a**–**e**) with the incorporation of the sulfonamide moiety for prospective employment in cancer therapeutics. The interaction of 4-((2, 4-dihydroxyphenyl)diazenyl) benzenesulfonamide **2a** with the appropriate cyanoacrylates **3a**–**g** in a 1:1 molar ratio in refluxing ethanol in the presence of a catalytic amount of piperidine generated the target 4*H*-chromene-3-carboxylates **7a**–**g**. Meanwhile, molecule **2a** interacted with 2-cyano-3-(4-oxo-4*H*-chromen-3-yl)acrylate **5** and afforded the ethyl 2′-amino-7′-hydroxy-4-oxo-6′-((4-sulfamoylphenyl)diazenyl)-4*H*,4′*H*-[3,4′-bichromene]-3′-carboxylate **8**. Additionally, the 4*H*-chromene-3-carboxylates **11a**–**e** were generated through the reaction of cyanoacrylate **3b** with molecules **2a**–**e** (1:2 molar ratio) in refluxing ethanol in the presence of piperidine. The structural frameworks of the composed hybrid compounds were recognized and established by utilizing analytical and spectroscopic data. The new series of sulfa azo 4*H*-chromene esters (**7a**–**g**, **8**, and **11a**–**e**) were adapted and assessed for their inhibitory behavior against the HCT-116, MCF-7, and HepG-2 tumor cell lines, targeting EGFR, MMP-2, and Carbonic anhydrase CAII. The 4*H*-chromene derivatives exhibited better antiproliferative activity in comparison with the sulfa and azo compounds. Most of the chromene/sulfonamide compounds displayed robust antiproliferative activity and demonstrated increased potency against the three malignant cell lines compared to the reference drugs Doxorubicin and Cisplatin. The molecular hybridization of the sulfa moiety to the azo phenol and the final formation of the 4*H*-chromene analogs led to the discovery of a new class of potent anticancer agents. Particular pathways were investigated, such as the EGFR and MMP-2 inhibitory analyses, confirming the presence of submicromolar inhibitory concentrations against EGFR, but moderate to low activity against hCAII. Conclusively, the molecular docking illustrated that the investigated derivatives bind in a similar manner as that of the reference inhibitor.

## Figures and Tables

**Table 1:** Cytotoxicity of the **7a**–**g**, **8**, and **11a**–**e** molecules against the three different cancer cell lines and normal cell line against **11a** and **11c**–**e** as a representative example.

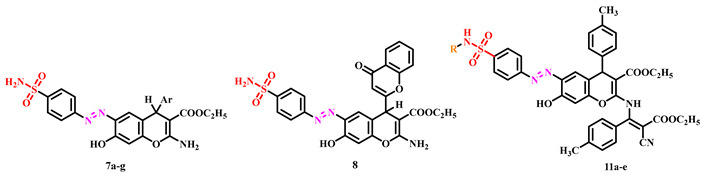										
Cpds.	Ar	IC_50_ (µM)			Cpds.	R	IC_50_ (µM)			Vero. CCL-81
		HepG-2	MCF-7	HCT-116			HepG-2	MCF-7	HCT-116	
**7a**	C_6_H_5_-	3.92 ±0.09	13.05 ±0.12	6.82 ±0.19	**8**		78.00 ±3.75	107.88 ±3.41	64.44 ±2.51	-
**7b**	4-CH_3_C_6_H_4_	45.66 ±2.88	29.52 ±3.29	17.28 ±4.29	**11a**	H	32.17 ±1.79	20.80 ±1.38	12.17 ±0.96	138.69 ±5.26
**7c**	4-OCH_3_C_6_H_4_	51.81 ±1.02	103.62 ±2.65	70.80 ±1.85	**11b**	COCH_3_	146.78 ±6.41	66.05 ±3.91	39.31 ±2.63	-
**7d**	4-NO_2_C_6_H_4_	34.89 ±2.79	97.90 ±3.08	113.82 ±5.34	**11c**	NH_2_-C=NH-	73.78 ±2.89	105.24 ±5.78	79.55 ±3.65	327.65 ±8.26
**7e**	4-FC_6_H_4_	2.92 ±3.52	6.15 ±1.23	8.00 ±3.45	**11d**	2-Thiazolyl	4.71 ±0.47	9.13 ±0.93	6.32 ±0.68	278.49 ±6.45
**7f**	4-ClC_6_H_4_	1.74 ±0.07	3.93 ±0.09	3.57 ±0.11	**11e**	3-methylisoxazol-5-yl	17.33 ±1.06	23.56 ±2.05	18.45 ±1.19	162.09 ±6.48
**7g**	2,4-Cl_2_C_6_H_3_	1.63 ±2.36	1.72 ±2.5	1.74 ±4.06	Cisplatin		10.93 ±0.19	14.66 ±0.03	8.36 ±0.07	-
Doxorubicin		0.66 ±0.02	0.64 ±0.03	0.90 ±0.07						

**Table 2:** The binding-affinity for compounds **7a**–**g**, **8**, and **11a**–**e** with docking score against EGFR, MMP-2, and hCAII.

No.	Δ*G*	RMSD	H.B.	EInt.	Eele	Ki	LE	No.	Δ*G*	RMSD	H.B.	EInt.	Eele	Ki	LE
4HJO															
**7a**	−7.844	1.909	−41.488	−20.541	−10.914	1.80	1.936	**8**	−9.153	1.241	−157.050	−22.427	−10.489	0.20	1.885
**7b**	−8.397	1.061	−42.880	−12.907	−10.745	0.70	1.562	**11a**	−9.985	1.420	−258.370	−32.923	−9.602	0.05	2.029
**7c**	−8.362	1.555	−38.642	−21.377	−14.727	0.74	0.155	**11b**	−9.661	1.815	−100.318	−19.416	−8.956	0.08	1.767
**7d**	−7.710	1.125	−21.609	−14.687	−11.687	2.22	1.815	**11c**	−10.003	1.110	−94.361	−13.177	−7.959	0.05	1.412
**7e**	−7.869	1.437	−33.456	−16.895	−10.659	1.70	1.432	**11d**	−6.420	1.095	−15.427	−15.208	−11.841	19.70	2.471
**7f**	−8.616	1.989	−26.329	−11.446	−11.836	0.48	1.486	**11e**	−7.068	1.025	−115.861	−18.606	−12.206	6.59	1.980
**7g**	−8.567	1.766	−65.246	−19.407	−10.628	0.52	0.941	**Erlotinib**	−5.361	1.342	−8.175	−17.976	−9.201	117	2.885
1HOV															
**7a**	−7.89	1.20	−55.10	−16.69	−11.53	1.64	1.73	**8**	−8.62	1.99	−26.33	−11.45	−11.84	0.48	0.94
**7b**	−7.88	1.60	−42.33	−18.56	−13.35	1.67	1.94	**11a**	−8.57	1.77	−65.25	−19.41	−10.63	0.52	1.88
**7c**	−7.84	1.91	−41.49	−20.54	−10.91	1.79	1.56	**11b**	−9.15	1.24	−157.05	−22.43	−10.49	0.20	2.03
**7d**	−8.40	1.06	−42.88	−12.91	−10.74	0.70	0.16	**11c**	−9.98	1.42	−258.37	−32.92	−9.60	0.05	1.77
**7e**	−8.36	1.55	−38.64	−21.38	−14.73	7.44	1.81	**11d**	−9.66	1.81	−100.32	−19.42	−8.96	0.08	1.41
**7f**	−7.71	1.13	−21.61	−14.69	−11.69	2.23	1.43	**11e**	−7.62	1.09	−20.33	−14.45	−2.84	2.59	1.94
**7g**	−7.87	3.44	−33.46	−16.90	−10.66	1.70	1.49	**hydroxamic**	−10.00	1.11	−94.36	−13.18	−7.96	0.05	1.36
3M04															
**7a**	−6.13	1.10	−41.07	−16.37	−9.33	32.0	1.94	**8**	−9.15	1.16	−68.16	−8.57	−9.33	0.20	1.88
**7b**	−7.99	1.19	−46.20	−8.34	−8.97	1.39	1.56	**11a**	−7.72	1.40	−148.09	−16.05	−7.05	2.19	2.03
**7c**	−7.53	1.81	−50.11	−9.56	−8.91	3.02	0.16	**11b**	−8.74	1.52	−278.47	−17.12	−7.32	0.39	1.77
**7d**	−7.60	1.61	−54.26	−9.04	−9.56	2.68	1.81	**11c**	−8.35	1.34	−95.54	−14.14	−7.26	0.76	1.41
**7e**	−7.35	1.98	−24.32	−11.12	−9.48	4.09	1.43	**11d**	−8.62	1.56	−80.25	−17.94	−6.22	0.48	1.36
**7f**	−7.50	1.68	−46.45	−9.36	−10.53	3.18	1.49	**11e**	−8.44	1.09	144.84	−18.19	−11.35	0.65	1.30
**7g**	−7.80	1.95	−53.97	−9.49	−10.03	1.91	0.94	**Sulfonamide**	−11.12	1.03	−123.16	−28.51	−16.33	0.01	2.33

Where, Δ*G*: Binding free energy of the ligand in Kcal/mol; RMSD: root mean square deviation; H.B.: H-bonding energy between protein and ligand in Kcal/mol; EInt.: Binding affinity of H-bond interaction with receptor in Kcal/mol; Eele: Electrostatic interaction over the receptor in Kcal/mol; LE: ligand efficacy in Kcal/mol; and Ki: inhibition constant in µM.

## Data Availability

Required data are available in the Supplementary Materials section.

## Supplementary Materials

The following supporting information can be downloaded at: https://www.mdpi.com/article/10.3390/ijms242316716/s1.
