# Supplementary material for: Synthesis and Characterization of a New Class of Chromene-Azo Sulfonamide Hybrids as Promising Anticancer Candidates with the Exploration of Their EGFR, hCAII, and MMP-2 Inhibitors Based on Molecular Docking Assays
Source: Int J Mol Sci. 2023 Nov 24;24(23):16716. doi: 10.3390/ijms242316716 (PMC10706804; doi:10.3390/ijms242316716)

Synthesis and Characterization of a New Class of Chromene-Azo Sulfonamide Hybrids as Promising Anticancer Candidates with the Exploration of their EGFR, *h*CAII and MMP-2 Inhibitors based Molecular Docking Assays

Fawzia F. Alblewia, Mosa H. Alsehli, Zainab M. Hritani, Areej A. Eskandrani, Wael H. Alsaedi, Majed O. Alawad, Ahmed A. Elhenawy, Hanaa Y. Ahmed, Mohamed S.A. El-Gaby, Tarek H. Afifi* and Rawda M. Okasha *

Supplementary Materials

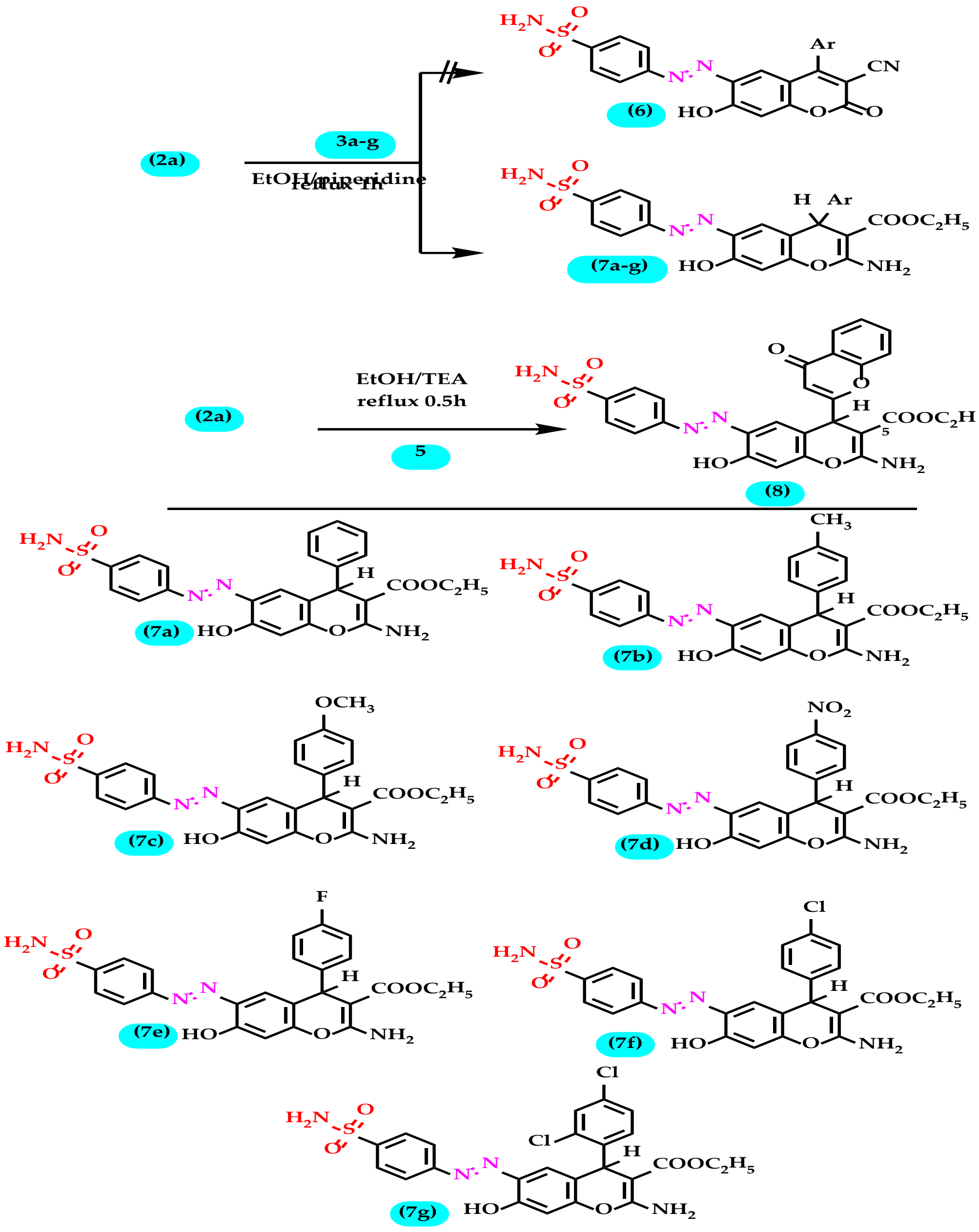

Scheme S1. Synthesis of 4-((2-amino-4-aryl-7-hydroxy-4-aryl-4*H*-chromen-6-yl)diazenyl]benzenesulfonamides **7a-g** and ethyl 2'-amino-7'-hydroxy-4-oxo-6'-((4-sulfamoyl phenyl) diazenyl)-4*H*,4'*H*-[3,4'-bichromene]-3'-carboxylate **8**.

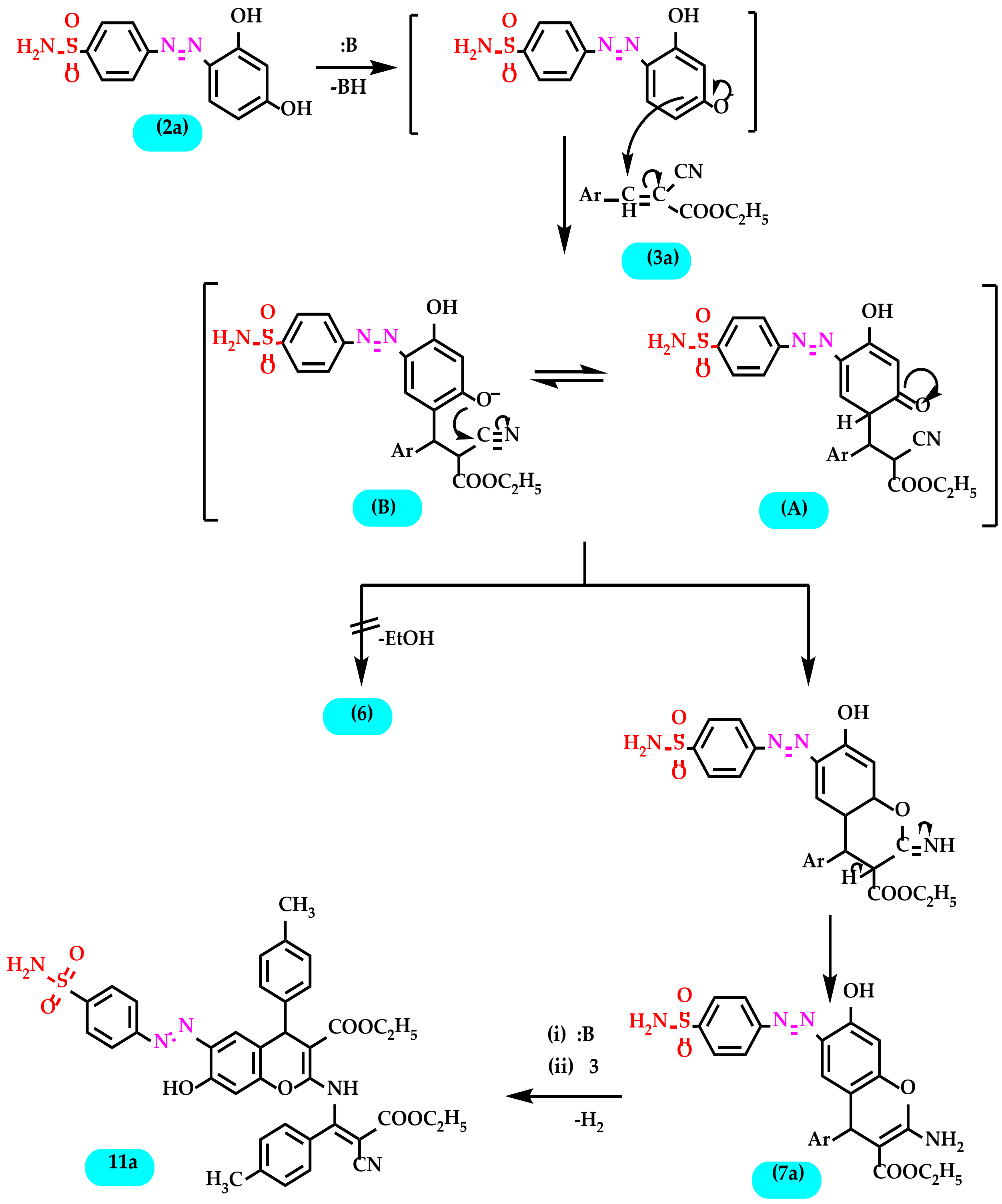

Scheme S2. The proposed mechanism of the formation of 4*H*-chromene-3-carboxylates **7a** and **9**.

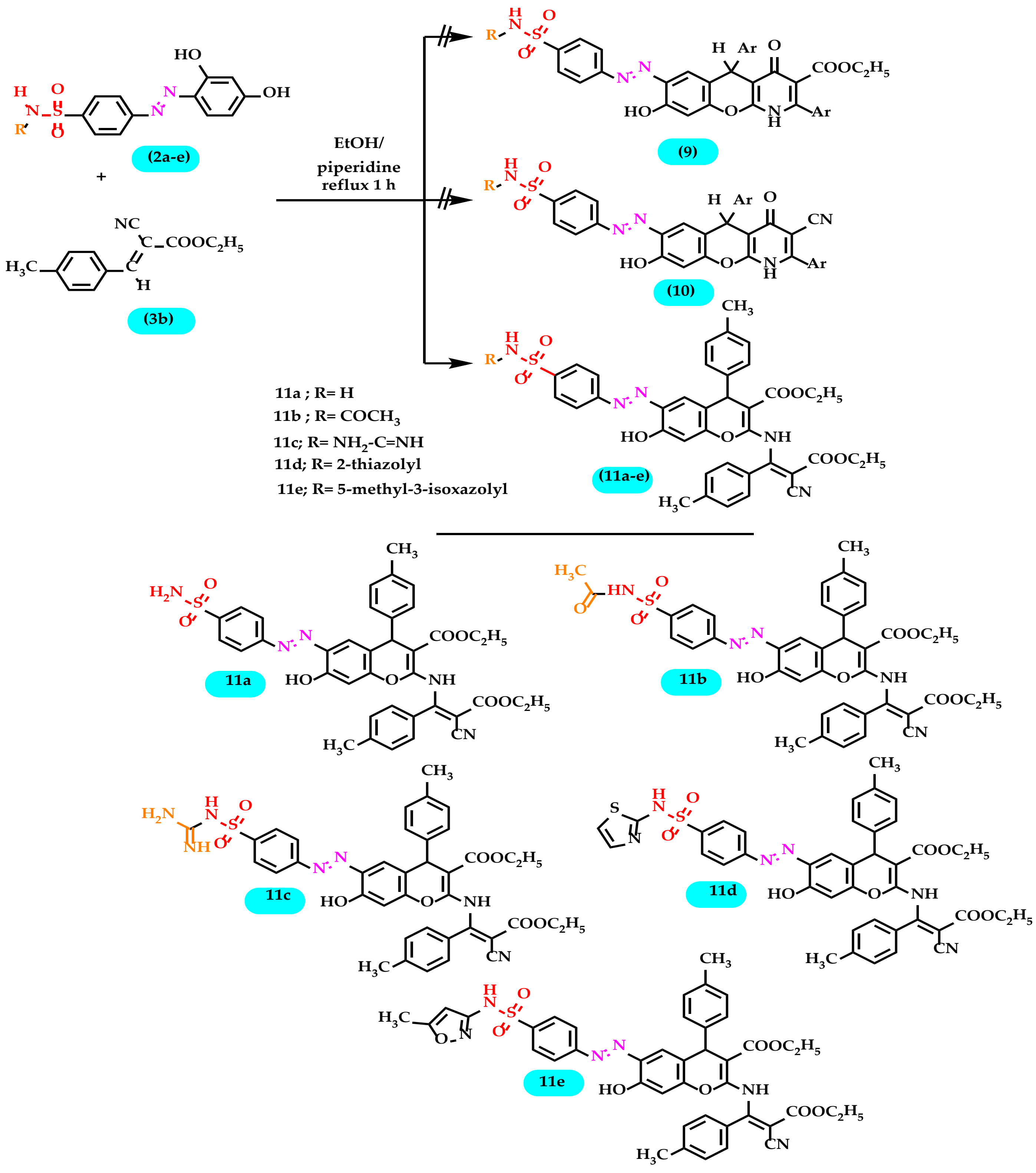

Scheme S3. Synthesis of 4-((2-amino-4-aryl-7-hydroxy-4-aryl-4*H*-chromen-6-yl)diazenyl]benzene sulfonamides **11a-e**

IR and NMR data for the investigated derivatives 7a-g, 8 and 11a-e

Ethyl 2-amino-7-hydroxy-4-phenyl-6-((4-sulfamoylphenyl)diazenyl)-4H-chromene-3-carboxylate 7a :

It was obtained in 86% yield as orange solid, m.p.: 229 ºC; IR: 3358 (NH/OH), 2983 (CH-aliph), 1673 (C=O), 1514 (N=N), 1168 (SO_2). ^1H NMR (DMSO-*d6*, ppm) δ: 1.29 (t, 3H, CH_3), 4.16 (2H, s, NH_2,exchangeable with D_2O), 4.19 (q, 2H, CH_2), 4.81 (s, 1H, pyran-4*H*), 6.72 (s, 1H, Ar-H), 6.83 (s, 2H, NH_2,exchangeable with D_2O), 7.30-7.41 (m, 5H, Ar-H), 7.82 (s, 1H, Ar-H), 8.08 (d, 2H, Ar-H), 8.14 (d, 2H, Ar-H), 10.29 (s, 1H, OH, exchangeable with D_2O); ^{13}C NMR (DMSO-*d6*, ppm) δ: 14.7 (CH_3), 35.7 (pyran-C4), 61.5 (CH_2), 84.8 (pyran-C3), 104.2, 113.9, 119.4, 128.5, 128.9, 128.9, 135.7, 141.1, 142.0, 151.1, 154.7, 157.6 (pyran-C2), 162.7, 166.4 (C=O). Anal. Calcd. for $C_{24}H_{22}N_4O_6S$; C, 58.29; H, 4.48; N, 11.33; S, 6.48. Found: 58.30; H, 4.40; N, 11.30; S, 6.40

Ethyl2-amino-7-hydroxy-6-((4-sulfamoylphenyl)diazenyl)-4-(p-tolyl)-4H-chromene-3-carboxylate 7b:

It was obtained in 82% yield as brown solid, m.p.: 239 ºC; IR: 3337 (NH/OH), 2985 (CH-aliph), 1666 (C=O), 1517 (N=N), 1071 (SO_2). ^1H NMR (DMSO-*d6*, ppm) δ: 1.16 (t, 3H, CH_3), 2.20 (s, 3H, CH_3), 4.01 (q, 2H, CH_2), 5.07 (s, 1H, pyran-4*H*), 6.83 (d, 1H, Ar-H), 7.02 (d, *J* = 7.84 Hz, 2H, Ar-H), 7.11 (d, *J* = 7.96 Hz, 2H, Ar-H), 7.52 (s, 2H, NH_2 ,exchangeable with D_2O), 7.68 (s, 2H, NH_2, exchangeable with D_2O), 7.75 (d, 1H, Ar-H), 7.97 d, *J* = 8.48 Hz,(d, *J* = 8.48 Hz, 2H, Ar-H), 8.13 (d, *J* = 8.56 Hz, 2H, Ar-H), 12.04 (s, 1H, OH, exchangeable with D_2O); ^{13}C NMR (DMSO-*d6*, ppm) δ: 14.8 (CH_3), 20.9 (CH_3), 34.2 (pyran-C4), 59.2 (CH_2), 77.4 (pyran-C3), 109.2, 116.4, 123.6, 124.2, 127.2, 128.1, 128.87, 135.5, 143.9, 145.8, 153.0, 153.6, 154.0, 160.6 (pyran-C2), 168.4 (C=O). Anal. Calcd. for $C_{25}H_{24}N_4O_6S$; C, 59.05; H, 4.76; N, 11.02; S, 6.30. Found: C, 59.00; H, 4.70; N, 11.00; S, 6.20.

Ethyl-2-amino-7-hydroxy-4-(4-methoxyphenyl)-6-((4-sulfamoylphenyl)diazenyl)-4H-chromene carboxylate 7c:

It was obtained in 85% yield as red solid, m.p.: 245 ºC; IR: 3335 (NH/OH), 2983 (CH-aliph), 1677 (C=O), 1504 (N=N), 1094 (SO_2). ^1H NMR (DMSO-*d6*, ppm) δ: 1.28 (t, 3H, CH_3), 3.78 (s, 3H, OCH_3) 4.17 (s, 2H, NH_2 ,exchangeable with D_2O), 4.19 (q, 2H, CH_2), 4.79 (s, 1H, pyran-4H), 6.72 (s, 1H, Ar-H), 6.78 (s, 2H, NH_2 ,exchangeable with D_2O), 6.90 (d, 2H, Ar-H), 7.38 (d, 2H, Ar-H), 7.83 (s, 1H, Ar-H), 8.08 (d, 2H, Ar-H), 8.14 d,(d, 2H, Ar-H), 10.30 (s, 1H, OH ,exchangeable with D_2O); ^{13}C NMR (DMSO-*d6*, ppm) δ: 14.7 (CH_3), 35.7 (pyran-C4), 56.0 (OCH_3), 61.5 (CH_2), 84.8 (pyran-C3), 104.2, 113.9, 114.0, 119.4, 123.8, 128.5, 129.2, 133.0, 135.7, 141.1, 151.1, 154.7, 157.6 (pyran-C2), 158.6, 162.7, 166.4 (C=O). Anal. Calcd. for $C_{25}H_{24}N_4O_7S$; C, 57.24; H, 4.61; N, 10.68; S, 6.11. Found: C, 57.20; H, 4.60; N, 10.60; S, 6.11.

Ethyl-2-amino-7-hydroxy-4-(4-nitrophenyl)-6-((4-sulfamoylphenyl)diazenyl)-4H-chromene-3-carboxylate 7d:

It was obtained in 81% yield as brown solid, m.p.: 240 ºC. IR: 3363 (NH/OH), 2983 (CH-aliph),1675 (C=O), 1468 (N=N), 1085 (SO_2). ^{1}H NMR (DMSO-d_6, ppm) δ: 1.13 (t, 3H, CH_3), 4.03 (q, 2H, CH_2), 5.23 (s, 1H, pyran-4H), 6.86 (d, 1H, Ar-H), 7.48-7.52 (m, 4H, Ar-H& NH_2), 7.76- 7.83 (m, 3H, Ar-H& NH_2), 7.97 (d, 2H, Ar-H), 8.14 (d, 4H, Ar-H), 12.04 (s, 1H, OH, exchangeable with D_2O); ^{13}C NMR (DMSO-d_6, ppm) δ: 14.7 (CH_3), 35.3 (pyran-C4), 59.5 (CH_2), 76.0 (pyran-C3),109.0, 114.5, 119.4, 123.4, 123.8, 127.3, 129.6, 135.5, 145.8, 146.2, 152.9, 153.3, 154.2, 154.4, 160.6 (pyran-C2), 168.1 (C=O). Anal. Calcd. for $C_{24}H_{21}N_5O_8S$; C, 53.43; H, 3.92; N, 12.98; S, 5.94. Found: C, 53.40; H, 3.90; N, 12.90; S, 5.94.

Ethyl-2-amino-4-(4-fluorophenyl)-7-hydroxy-6-((4-sulfamoylphenyl)diazenyl)-4H-chromene-3-carboxylate 7e

It was obtained in 82% yield as brown solid, m.p.: 219 ºC; IR: 3327 (NH/OH), 2983 (CH-aliph), 1675 (C=O), 1500 (N=N), 1346, 1073 (SO_2). ^{1}H NMR (DMSO-d_6, ppm) δ: 1.36 (t, 3H, CH_3), 4.16 (s, 2H, NH_2 ,exchangeable with D_2O), 4.18 (q, 2H, CH_2), 4.92 (s, 1H, pyran-4H), 6.73 (s, 1H, Ar-H), 7.02 (t, 2H, Ar-H), 7.38 (t, 2H, Ar-H), 7.79-7.80 (bs, 3H, Ar-H& NH_2), 8.08 (d, 2H, Ar-H), 8.14 (d, 2H, Ar-H), 10.30 (s, 1H, OH ,exchangeable with D_2O); ^{13}C NMR (DMSO-d_6, ppm) δ: 14.7 (CH_3), 35.7 (pyran-C4), 61.5 (CH_2), 84.8 (pyran-C3), 104.2, 113.9, 114.5, 119.4, 123.8, 128.5, 129.0, 135.7, 137.1, 141.1, 151.1, 154.7, 157.6 (pyran-C2), 162.7, 163.1, 166.4 (C=O). Anal. Calcd. for $C_{24}H_{21}FN_4O_6S$; C, 56.25; H, 4.13; N, 10.93; S, 6.26. Found: C, 56.20; H, 4.10; N, 10.90; S, 6.20

Ethyl-2-amino-4-(4-chlorophenyl)-7-hydroxy-6-((4-sulfamoylphenyl)diazenyl)-4H-chromene-3-carboxylate 7f :

It was obtained in 85% yield as light brown solid, m.p.: 234 ºC; IR: 3365 (NH/OH), 2983 (CH-aliph), 1668 (C=O), 1515 (N=N), 1150 (SO_2). ^{1}H NMR (DMSO-d_6, ppm) δ: 1.16 (t, 3H, CH_3), 4.03 (q, 2H, CH_2), 5.09 (s, 1H, pyran-4H), 6.84 (d, 1H, Ar-H), 7.23 (d, J = 8.36 Hz, 2H, Ar-H), 7.30 (d, J = 8.28 Hz, 2H, Ar-H), 7.52 (s, 2H, NH_2 ,exchangeable with D_2O), 7.74- 7.77 (m, 3H, Ar-H and NH), 7.97 (d, J = 8.40 Hz, 2H, Ar-H), 8.14 (d, J = 8.36 Hz, 2H, Ar-H), 12.02 (s, 1H, OH ,exchangeable with D_2O); ^{13}C NMR (DMSO-d_6, ppm) δ: 14.5 (CH_3), 34.3 (pyran-C4), 59.5 (CH_2), 76.9 (pyran-C3), 108.8, 115.5, 123.6, 124.0, 127.1, 127.9, 129.9, 130.9, 135.3, 145.6, 152.6, 153.5, 154.0, 160.6 (pyran-C2), 168.2 (C=O). Anal. Calcd. for $C_{24}H_{21}ClN_4O_6S$; C, 54.50; H, 4.00; N, 10.59; S, 6.06. Found: C, 54.50; H, 4.00; N, 10.50; S, 6.00

Ethyl-2-amino-4-(2,4-dichlorophenyl)-7-hydroxy-6-((4-sulfamoylphenyl)diazenyl)-4H-chromene-3-carboxylate 7g:

It was obtained in 80% yield as orange solid, m.p.: 259 ºC; IR: 3246 (NH/OH), 2983 (CH-aliph), 1669 (C=O), 1464 (N=N), 1140 (SO_2). ^{1}H NMR DMSO-d_6, ppm) δ: 1.32 (t, 3H, CH_3), 4.14 (s, 2H, NH_2,exchangeable with D_2O), 4.24 (q, 2H, CH_2), 4.98 (s, 1H, pyran-4H), 5.57 (s, 1H, OH, exchangeable with D_2O), 6.47 (s, 1H, Ar-H), 7.24 (d, 1H, Ar-H), 7.39 (m, 1H, Ar-H), 7.45 (d, 1H, Ar-H), 7.82 (s, 1H, Ar-H), 8.09- 8.15 (dd, 4H, Ar-H), 9.27 (s, 2H, NH_2,exchangeable with D_2O); ^{13}C NMR (DMSO-d_6, ppm) δ: 14.7 (CH_3), 40.2 (pyran-C4), 61.5 (CH_2), 84.6 (pyran-C3), 103.4, 113.4, 119.4, 124.1, 128.5, 128.8, 130.9, 133.3, 134.7, 135.2, 135.5, 139.7, 141.1, 151.1, 155.3, 157.6

(pyran-C2), 162.5, 166.4 (C=O). Anal. Calcd. for $C_{24}H_{20}Cl_2N_4O_6S$; C, 51.16; H, 3.58; N, 9.94; S, 5.69. Found: C, 51.10; H, 3.50; N, 9.90; S, 5.60.

Ethyl 2-amino-7′-hydroxy-4-oxo-6′-((4-sulfamoylphenyl)diazenyl)-4H,4′H-[3,4′-bichromene]-3′-carboxylate 8.

To a mixture of compound **2a** (0.01 mol) and 2-cyano-3-(4-oxo-4*H*-chromen-3-yl) acrylate **5** (0.01 mol) in ethanol (15 ml), a few drops of triethylamine was added. The reaction mixture was refluxed for 0.5 h. The solid product, so formed, was collected by filtration and recrystallized from ethanol to yield **8**.

It was obtained in 81% yield as red solid, m.p.: 175 ºC. IR: 3300, 3114 (NH/OH), 2983 (CH-aliph), 1746, 1615 (C=O), 1502 (N=N), 1151 (SO_2). ^{1}H NMR (DMSO-d_6, ppm) δ: 1.21 (t, 3H, CH_3), 4.08 (q, 2H, OCH_2), 4.82 (s, 1H, pyran-4H), 6.41- 8.44 (m, 15H, Ar-H & NH_2), 10.80 (s, 1H, OH, exchangeable with D_2O); ^{13}C NMR (DMSO-d_6, ppm) δ: 19.6 (CH_3), 34.5 (pyran-C4), 63.8 (CH_2), 77.3 (pyran-C3), 108.2, 113.4, 114.8, 116.6, 127.2, 128.0, 132.1, 132.1, 137.9, 149.9, 150.3, 157.6, 157.7, 159.0, 159.5, 160.6, 162.7, 166.2, 169.2 (pyran-C2), 173.5, 180.6 (C=O). Anal. Calcd. for $C_{27}H_{22}N_4O_8S$; C, 57.65; H, 3.94; N, 9.96; S, 5.70. Found: C, 57.60; H, 3.90; N, 9.90; S, 5.70.

General procedure for the synthesis of ethyl 2-((2-cyano-3-ethoxy-3-oxo-1-(p-tolyl)prop-1-en-1-yl)amino)-7-hydroxy-6-((4-sulfamoylphenyl)diazenyl)4-(p-tolyl)-4H-chromene-3-carboxylates 11a-e :

A solution of α-Cyanoacrylates **3b** (0.02 mol) in ethanol (15 ml), compound **2** (0.01 mol), and a few drops of piperidine was refluxed for 1 h and cooled. The precipitate was filtrate off and crystallized from the appropriate solvent.

Ethyl 2-((2-cyano-3-ethoxy-3-oxo-1-(p-tolyl)prop-1-en-1-yl)amino)-7-hydroxy-6-((4-sulfamoyl-phenyl)diazenyl)-4-(p-tolyl)-4H-chromene-3-carboxylates 11a.

This compound was obtained as brown crystals from ethanol; yield 84%; m.p. 140-141 °C. IR: 3427 (NH/OH), 2983 (CH-aliph), 2217 (CN), 1722 (C=O), 1597 (N=N), 1346, 1152 (SO_2). ^{1}H NMR (DMSO-d_6, ppm) δ: 1.17 (t, 3H, CH_3), 1.30 (t, 3H, CH_3), 2.22,2.33 (2s, 6H, 2CH_3), 4.02 (q, 2H, CH_2), 4.31 (q, 2H, CH_2), 5.08 (s, 1H, pyran-4H), 6.96, 7.02 (2d, 4H, AB-system), 7.38, 7.94 (2d, 4H, AB-system), 8.02, 8.11 (2d, 4H, AB-system), 6.81 (d, 1H, Ar-H), 7.72 (d, 1H, Ar-H), 7.60 (s, 1H, NH, exchangeable with D_2O), 8.33 (s, 2H, NH_2, exchangeable with D_2O), 12.07 (s, 1H, OH, exchangeable with D_2O). Anal. Calcd. for $C_{38}H_{35}N_5O_8S$; C, 63.23; H, 4.89; N, 9.70; S, 4.44. Found: C, 63.20; H, 4.80; N, 9.70; S, 4.40

Ethyl 6-(4-(N-acetylsulfamoyl)phenyl)diazenyl)-2-(2-cyano-3-ethoxy-3-oxo-1-(p-tolyl)prop-1-en-1-yl)amino)-7-hydroxy-4-(p-tolyl)-4H-chromene-3-carboxylate 11b.

This compound was obtained as red crystals from ethanol; yield 86%; m.p. 90-91 °C. IR: 3427, (NH/OH), 2984 (CH-aliph), 2217 (CN), 1723, 1682 (2C=O), 1598 (N=N), 1376, 1143 (SO_2). ^{1}H NMR (DMSO-d_6, ppm) δ: 1.17 (t, 3H, CH_3), 1.30 (t, 3H, CH_3), 2.19 (2s, 6H, 2CH_3), 2.39 (s, 3H, CH_3), 4.01 (q, 2H, CH_2), 4.30 (q, 2H, CH_2), 5.07 (s, 1H, pyran-4H), 6.85, 7.72 (2d, 4H, AB-system), 7.00, 7.12 (2d, 4H, AB-system), 7.28, 7.94 (2d, 4H, AB-system), 7.62 (s, 1H, NH, exchangeable with D_2O), 7.72 (d, 1H,

Ar-H), 8.04 (d, 1H, Ar-H), 8.32 (s, 1H, NH, exchangeable with D_2O), 12.15 (s, 1H, OH, exchangeable with D_2O). Anal. Calcd. for $C_{40}H_{37}N_5O_9S$; C, 62.90; H, 4.88; N, 9.17; S, 4.20. Found: C, 62.90; H, 4.88; N, 9.17; S, 4.20.

Ethyl 6-(4-(N-carbamimidoylsulfamoyl)phenyl)diazenyl)-2-(2-cyano-3-ethoxy-3-oxo-1-(p-tolyl)prop-1-en-1-yl)amino)-7-hydroxy-4-(p-tolyl)-4H-chromene-3-carboxylate 11c.

This compound was isolated as brown crystals from 1,4-dioxane; yield 82%; m.p. 160-161 °C. IR: 3439, 3337, 3223 (OH /NH_2), 2980 (CH-aliph), 2223 (CN), 1721, 1682 (2C=O), 1624 (C=C), 1598 (N=N), 1400, 1136 (SO_2). ^{1}H NMR (DMSO-d_6, ppm) δ: 1.10 (t, 3H, CH_3), 1.36 (t, 3H, CH_3), 2.18, 2.36 (2s, 6H, 2CH_3), 4.01 (q, 2H, CH_2), 4.29 (q, 2H, CH_2), 5.08 (s, 1H, pyran-4H), 6.77 (br, 2H, NH_2, exchangeable with D_2O), 6.81, 7.35 (2d, 4H, AB-system), 6.99, 7.10 (2d, 4H, AB-system), 7.92, 8.01 (2d, 4H, AB-system), 7.60, 7.84, 8.30 (3s, 3H, 3NH, exchangeable with D_2O), 12.20 (s, 1H, OH, exchangeable with D_2O), 7.69 (d, 1H, Ar-H), 7.72 (d, 1H, Ar-H). Anal. Calcd. for $C_{39}H_{37}N_7O_8S$; C, 61.33; H, 4.88; N, 12.84; S, 4.20. Found: C, 61.33; H, 4.88; N, 12.84; S, 4.20.

Ethyl 2-(2-cyano-3-ethoxy-3-oxo-1-(p-tolyl)prop-1-en-1-yl)amino)-7-hydroxy-6-(4-(N-(thiazol-2-yl)sulfamoyl)phenyl)diazenyl)-4-(p-tolyl)-4H-chromene-3-carboxylate 11d.

This compound was isolated as brown crystals from ethanol; yield 87%; m.p. 130-131°C. IR: 3425, 3260 (OH/NH), 2979 (CH-aliph), 2222 (CN), 1721, 1681 (2C=O), 1620 (C=C), 1597 (N=N), 1327, 1143 (SO_2). ^{1}H NMR (DMSO-d_6, ppm) δ: 1.17 (t, 3H, CH_3), 1.31 (t, 3H, CH_3), 2.19, 2.40 (2s, 6H, 2CH_3), 4.02 (q, 2H, CH_2), 4.31 (q, 2H, CH_2), 5.01 (s, 1H, pyran-4H), 6.81, 6.84 (2d, 2H, thiazole-H), 6.85, 7.01 (2d, 4H, AB-system), 7.10, 7.73 (2d, 4H, AB-system), 7.95, 8.05 (2d, 4H, AB-system), 7.26 (d, 1H, Ar-H), 7.39 (d, 1H, Ar-H), 7.61, 7.84, 8.34 (2s, 2H, 2NH, exchangeable with D_2O), 12.12 (s, 1H, OH, exchangeable with D_2O). Anal. Calcd. for $C_{41}H_{36}N_6O_8S_2$; C, 61.18; H, 4.51; N, 10.44; S, 7.97. Found: C, 61.18; H, 4.51; N, 10.44; S, 7.97

Ethyl 2-(2-cyano-3-ethoxy-3-oxo-1-(p-tolyl)prop-1-en-1-yl)amino)-7-hydroxy-6-(4-(N-(5-methylisoxazol-3-yl)sulfamoyl)phenyl)diazenyl)-4-(p-tolyl)-4H-chromene-3-carboxylate 11e.

This compound was isolated as brown crystals from 1,4-dioxane; yield 89%; m.p. 140-141 °C. IR: 3450, 3240 (OH/NH), 2983 (CH-aliph), 2217 (CN), 1723, (C=O), 1615 (C=C), 1597 (N=N), 1394, 1154 (SO_2). ^{1}H-NMR (DMSO-d_6, ppm) δ: 1.05 (t, 3H, CH_3), 1.20 (t, 3H, CH_3), 2.21, 2.29, 2.36 (3s, 9H, 3CH_3), 3.43 (q, 2H, CH_2), 4.28 (q, 2H, CH_2), 5.05 (s, 1H, pyran-4H), 6.14 (d, 2H, isoxazloe-H), 6.53, 7.53 (2d, 4H, AB-system), 6.93, 6.98 (2d, 4H, AB-system), 7.35, 7.91 (2d, 4H, AB-system), 7.44 (d, 1H, Ar-H), 8.29 (d, 1H, Ar-H), 7.98, 8.29 (2s, 2H, 2NH, exchangeable with D_2O), 12.21 (s, 1H, OH, exchangeable with D_2O). Anal. Calcd. for $C_{42}H_{38}N_6O_9S$; C, 62.83; H, 4.77; N, 10.47; S, 3.99. Found: C, 62.83; H, 4.77; N, 10.47; S, 3.99.

IR and NMR Spectra for some selected derivatives

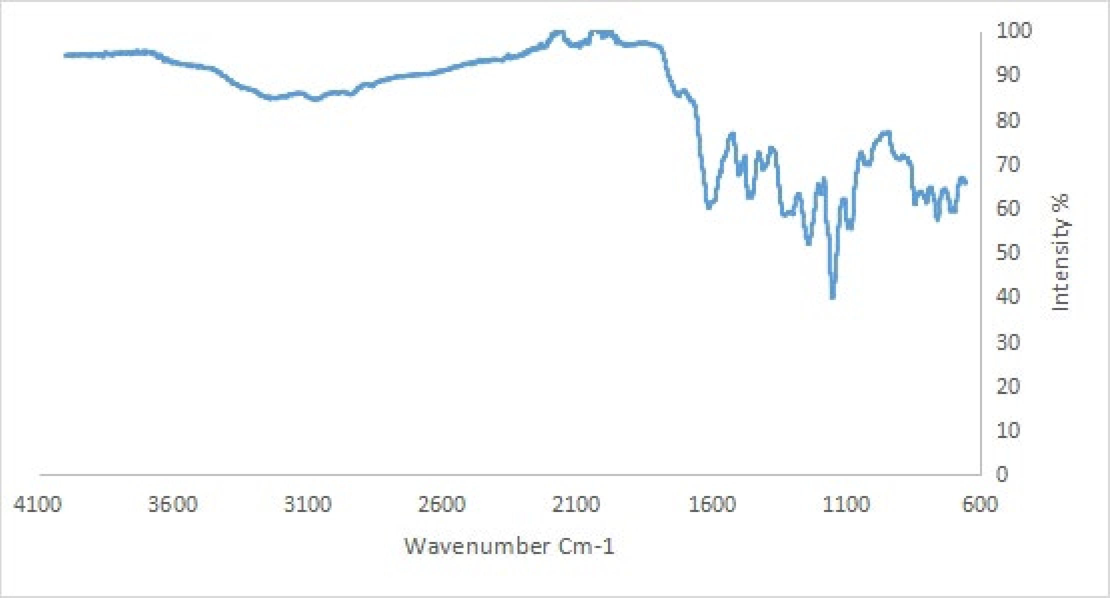

Figure S1: IR spectra of **8**.

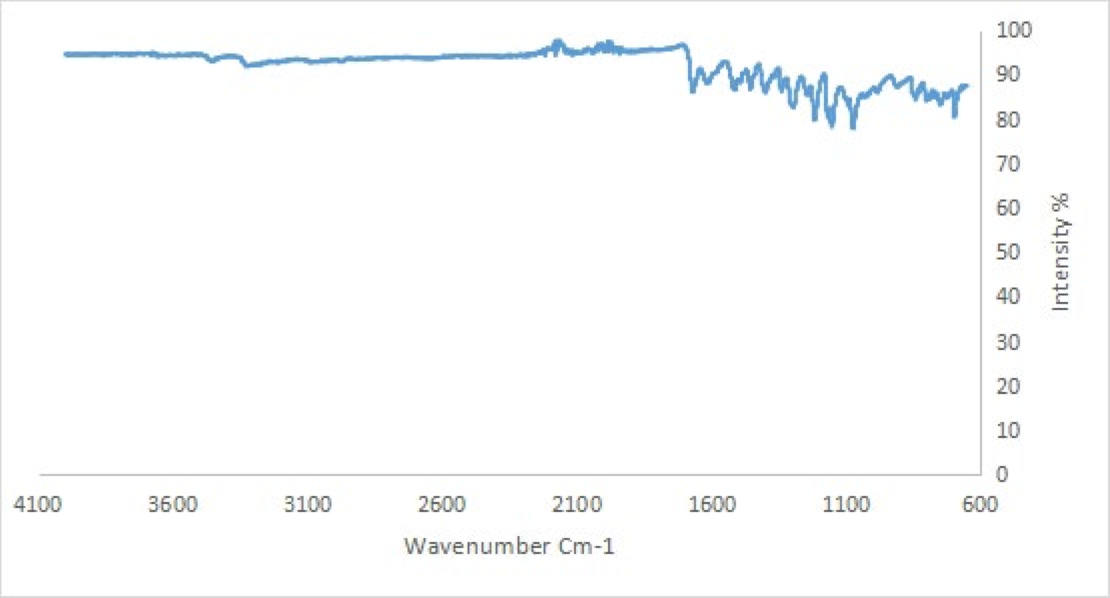

Figure S2: IR spectra of **7a**.

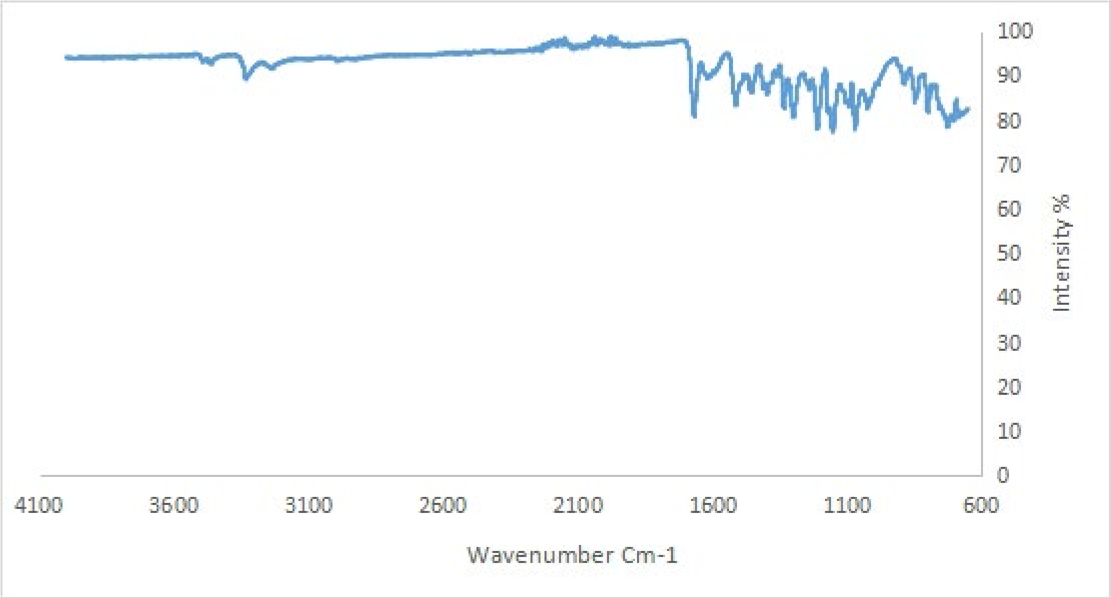

Figure S3: IR spectra of **7b**.

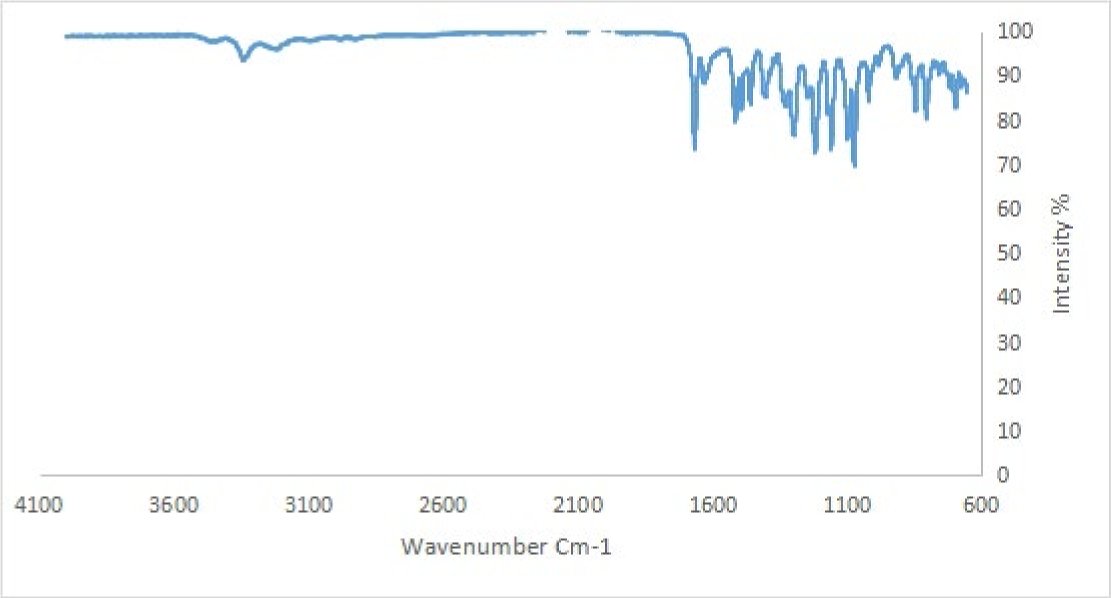

Figure S4: IR spectra of **7f**.

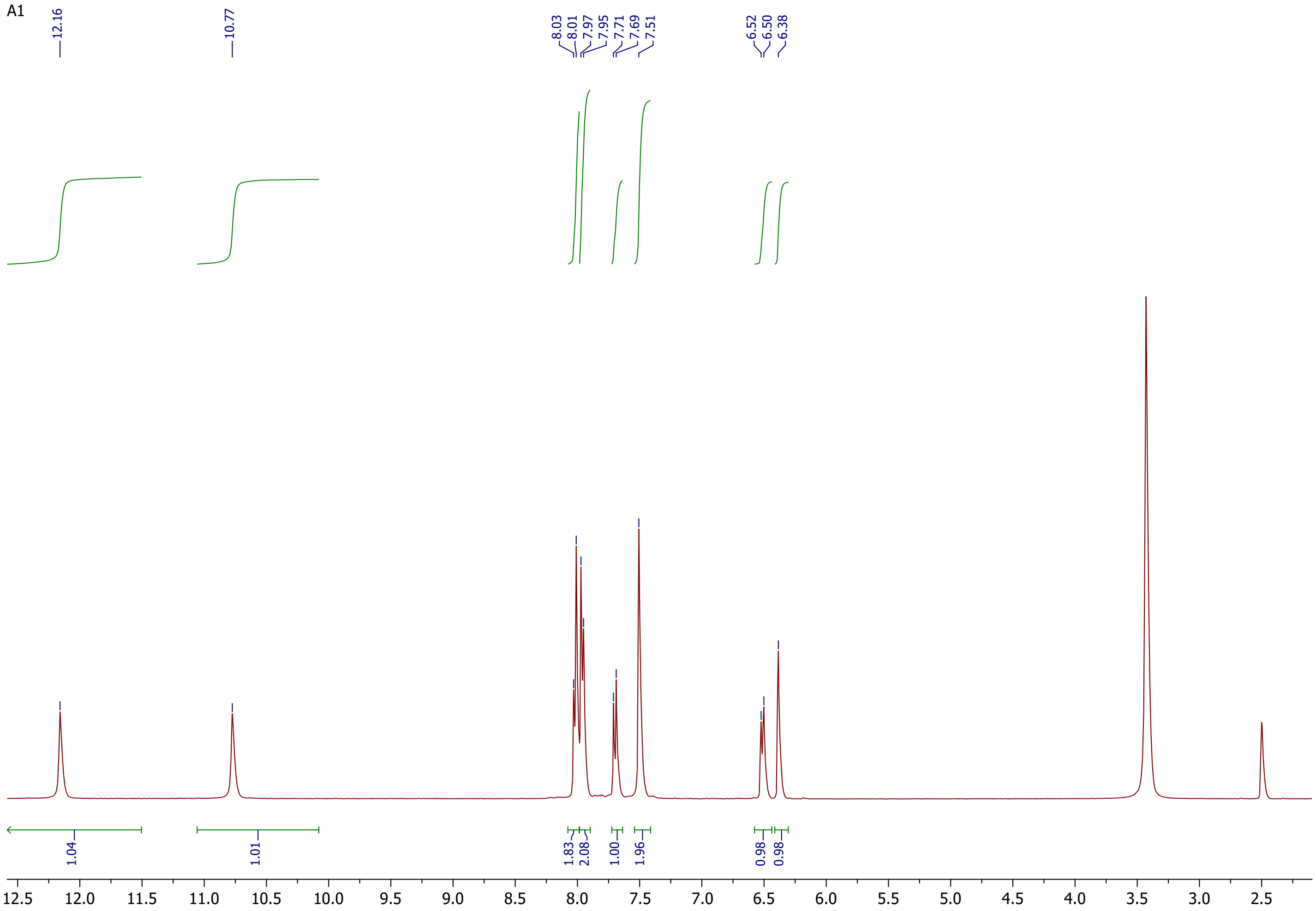

Figure S5: ^{1}H NMR spectra of azo dye **2a**

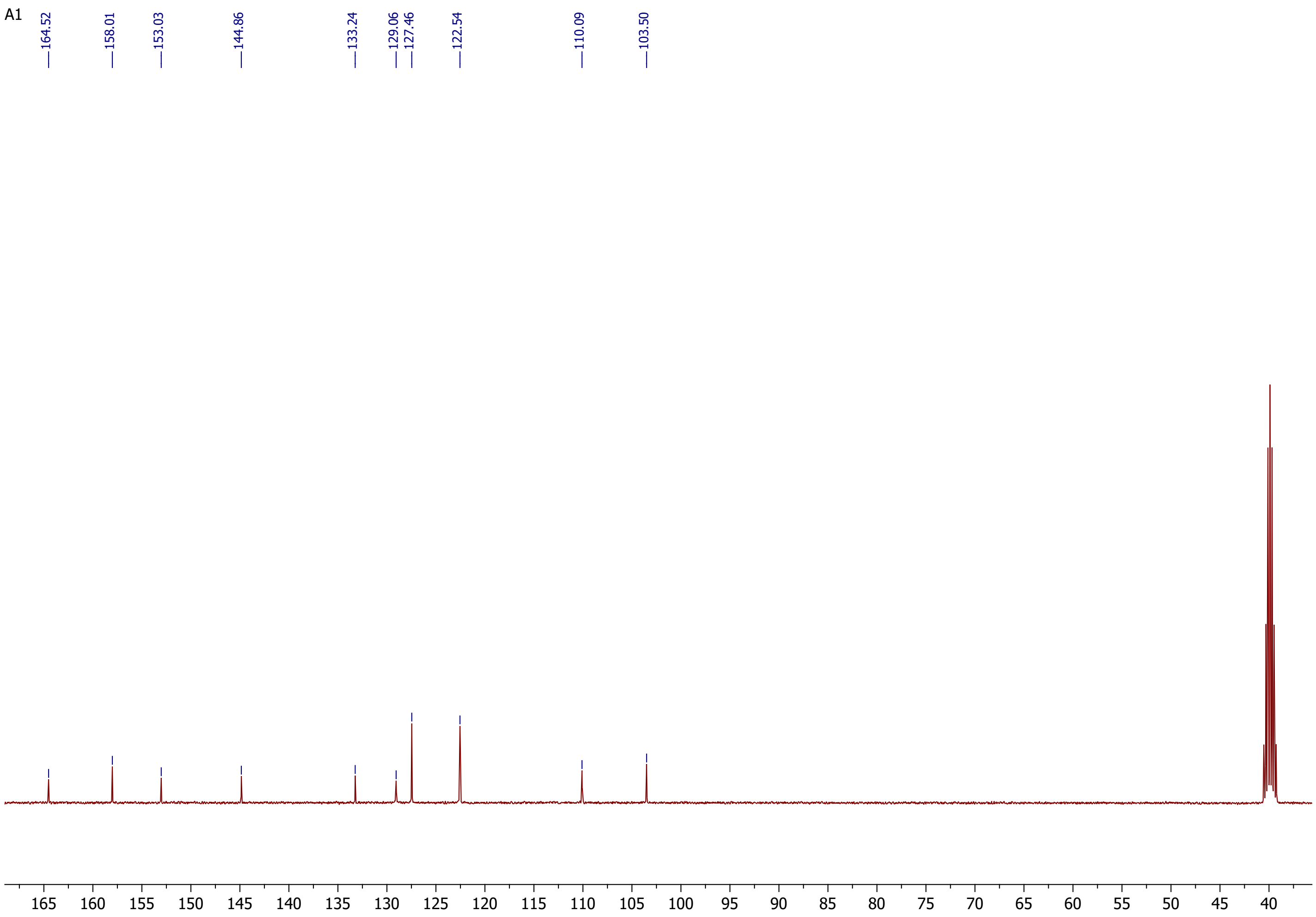

Figure S6: ^{13}C NMR spectra of azo dye **2a**

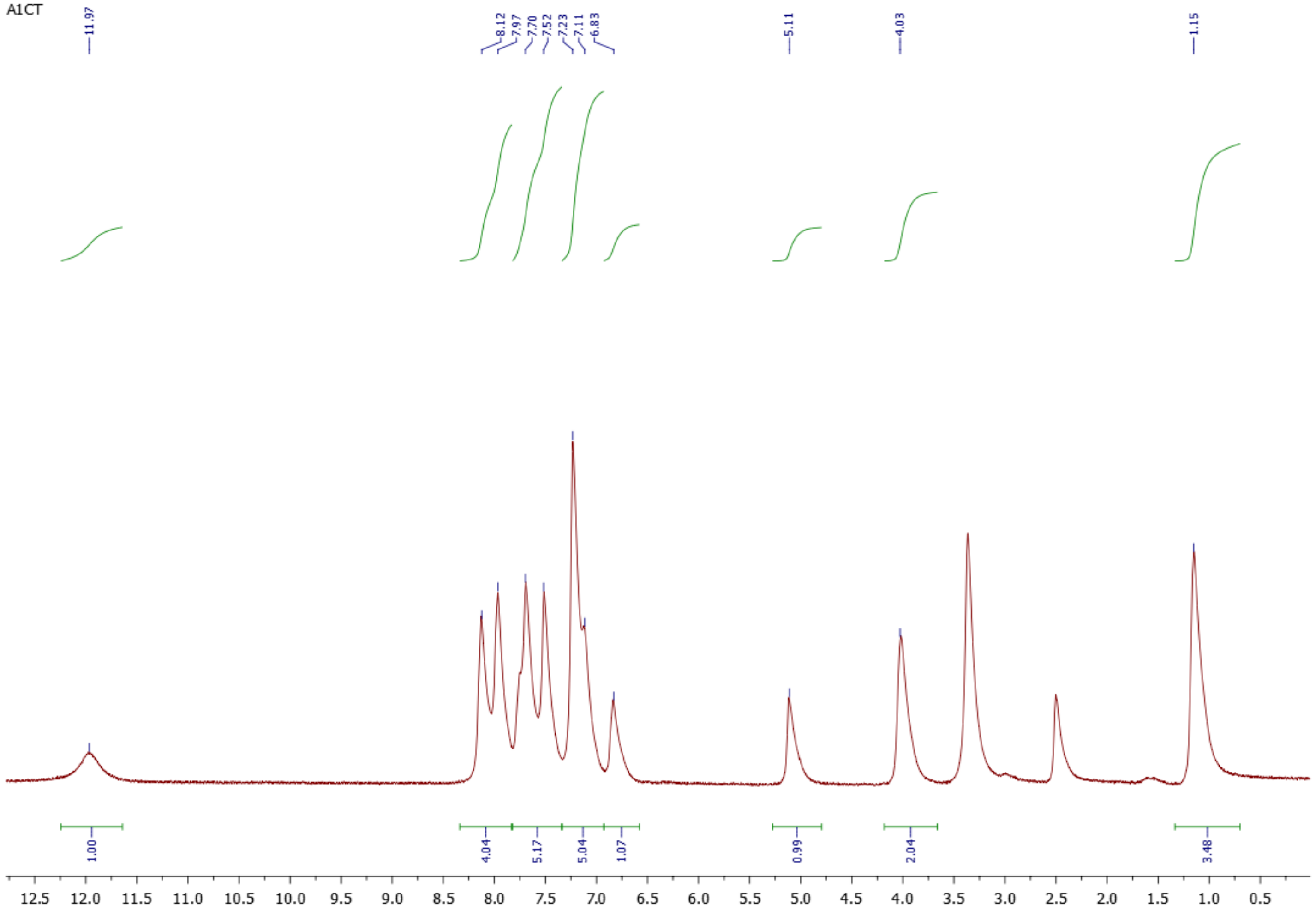

Figure S7: ^{1}H NMR spectra of azo sulfa chromene ester **7a**

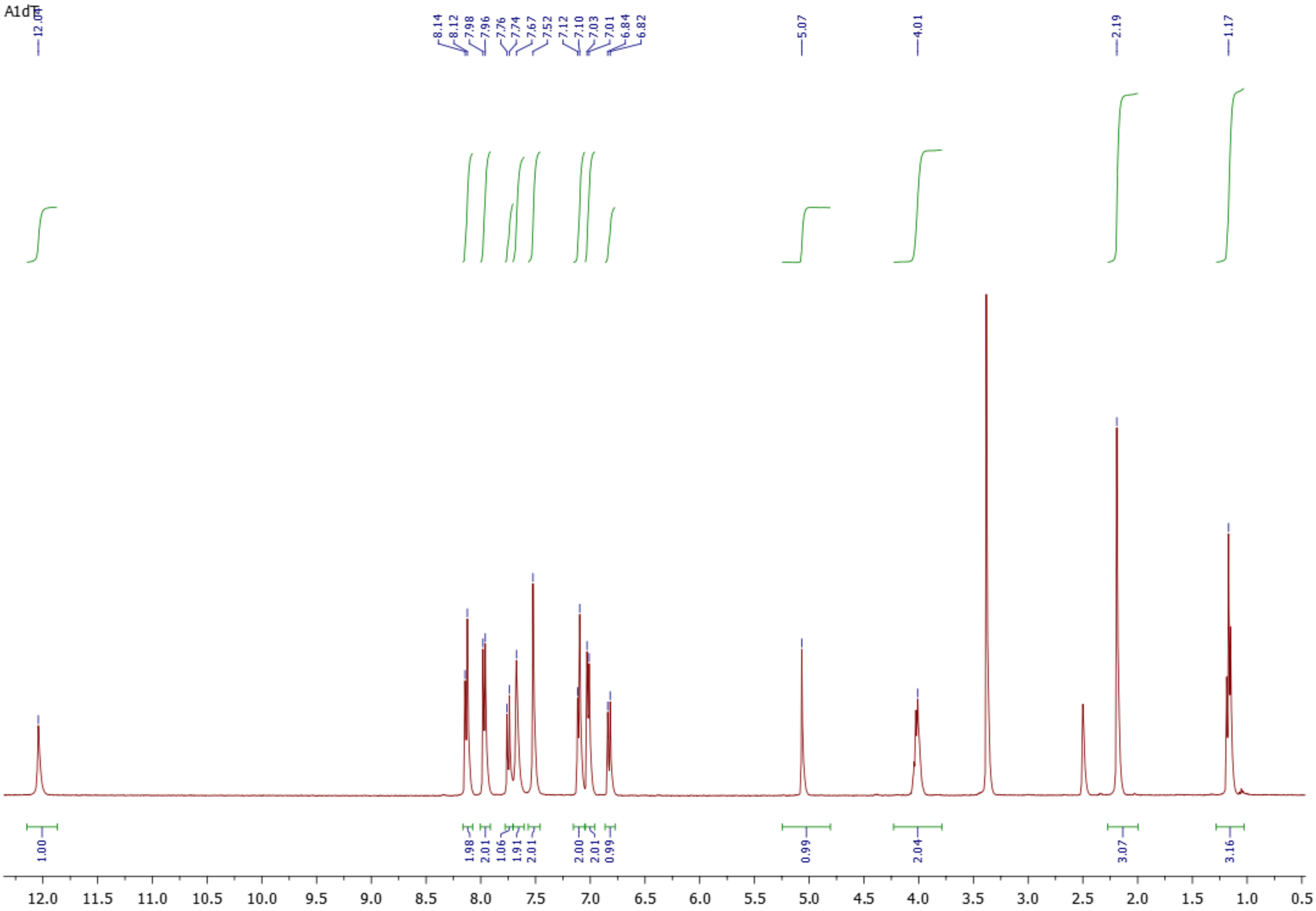

Figure S8: ^{1}H NMR spectra of azo sulfa chromene ester **7b**

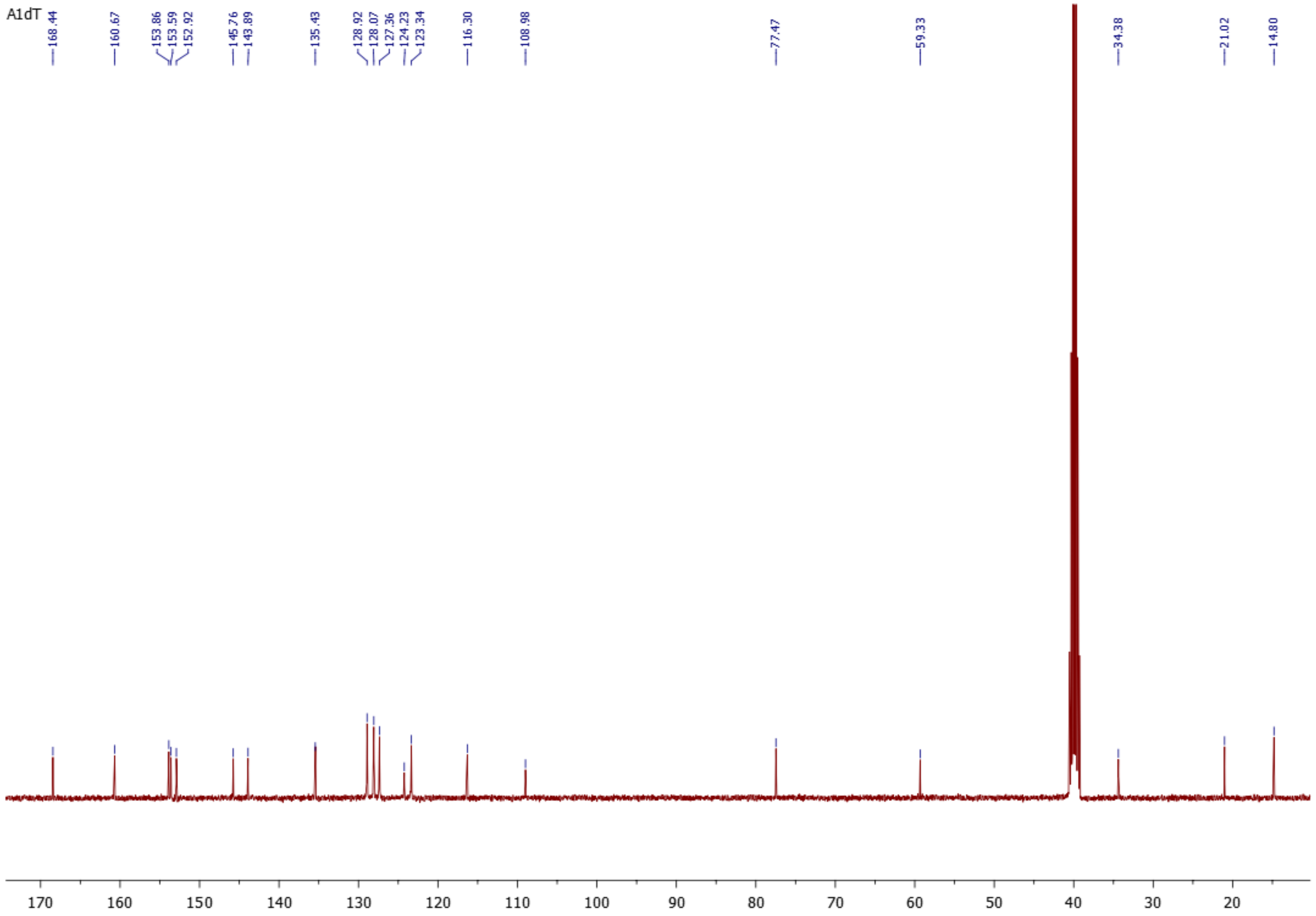

Figure S9: ^{13}C NMR spectra of azo dye **7b**

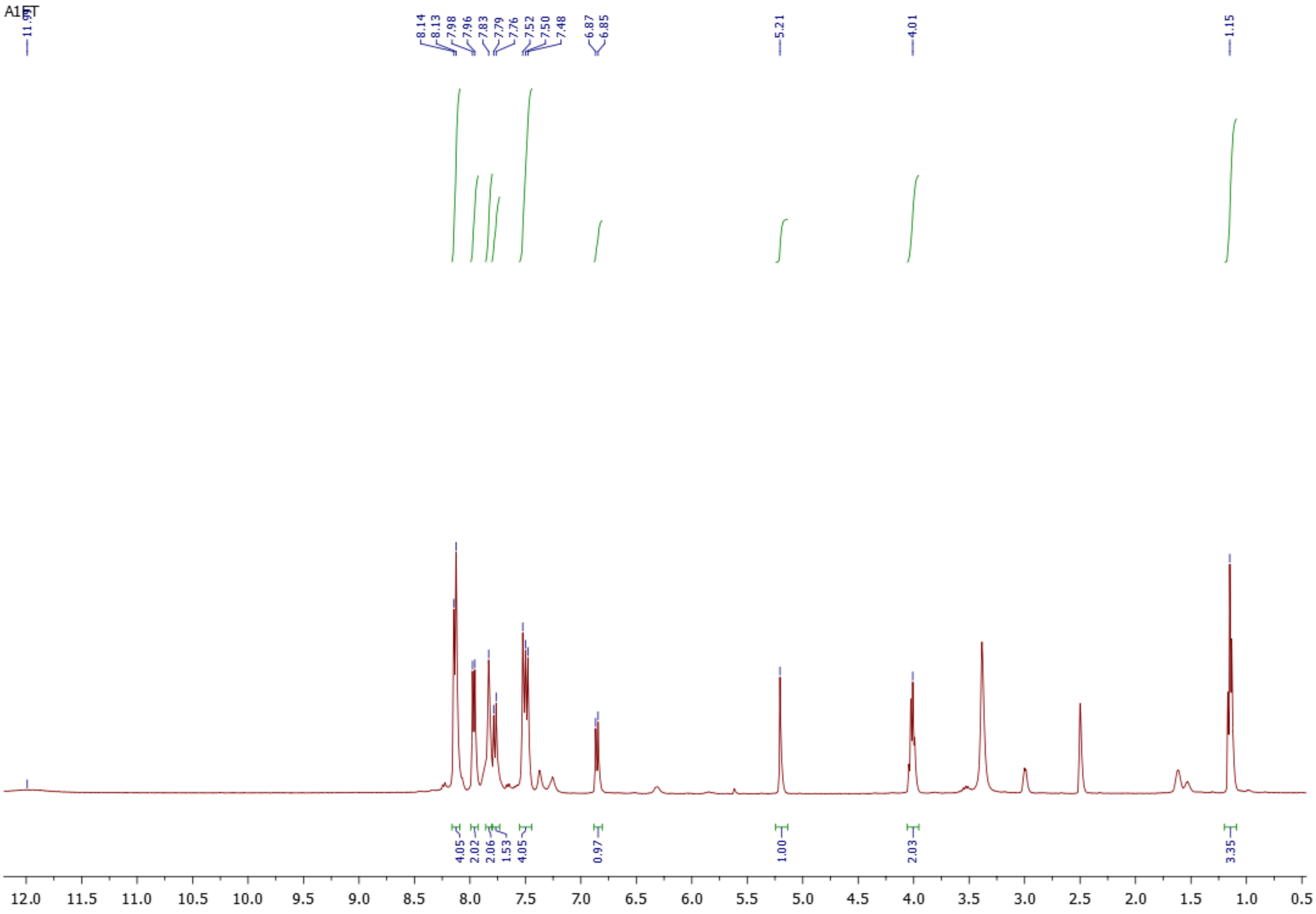

Figure S10: ^{1}H NMR spectra of azo sulfa chromene ester **7d**.

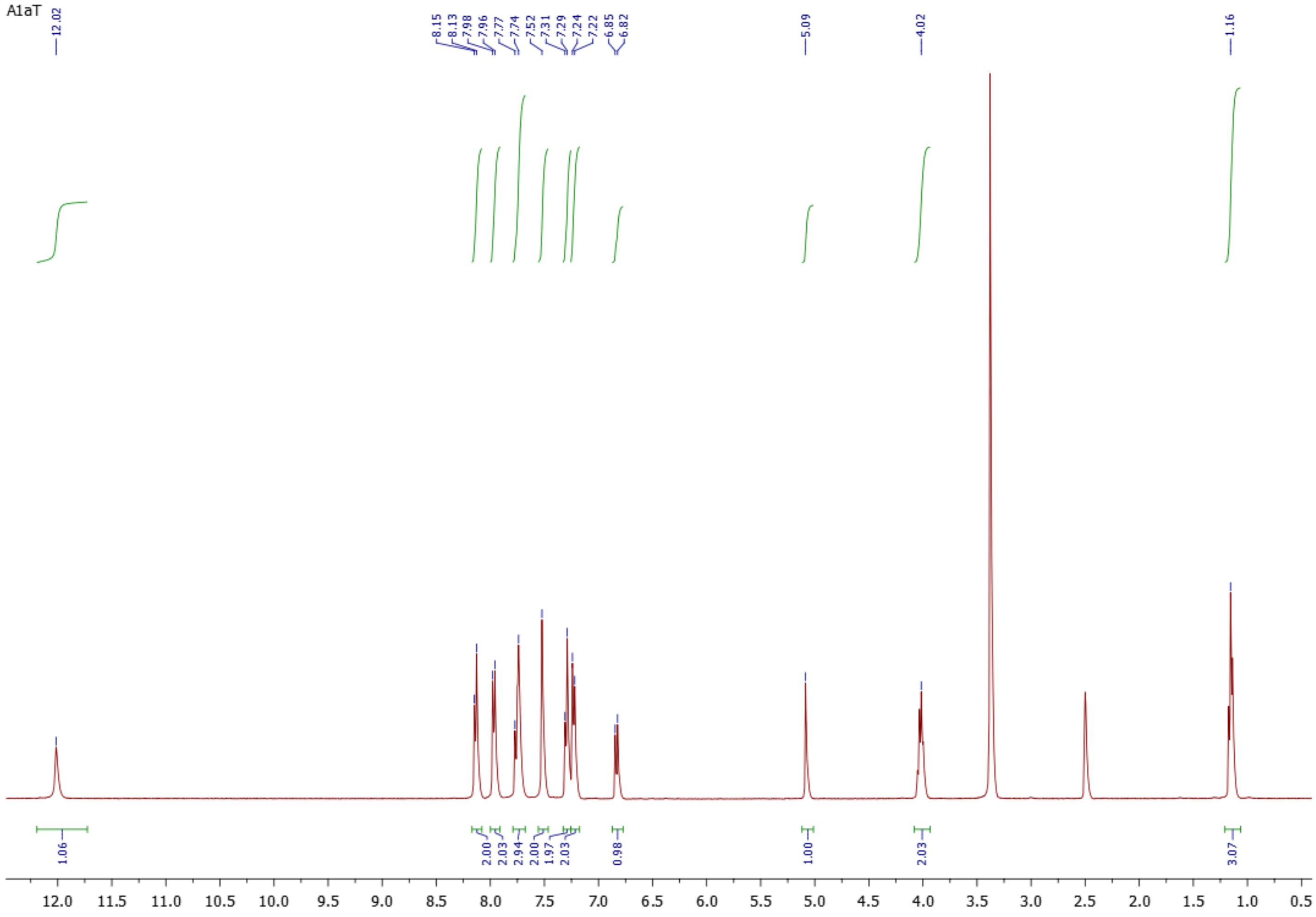

Figure S11: ^{1}H NMR spectra of azo sulfa chromene ester **7f**.

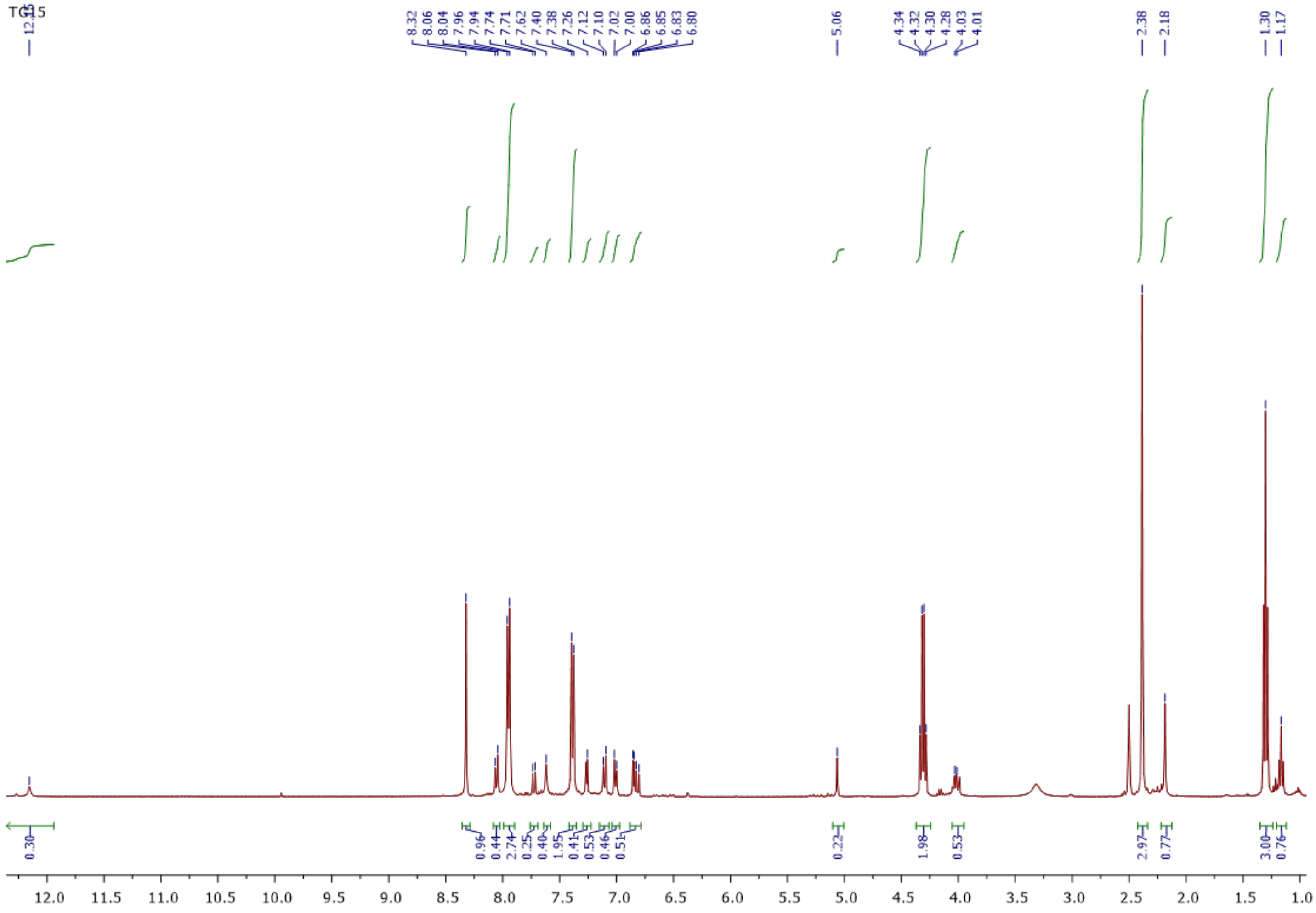

Figure S12: ^{1}H NMR spectra of azo sulfa chromene ester **11b**.

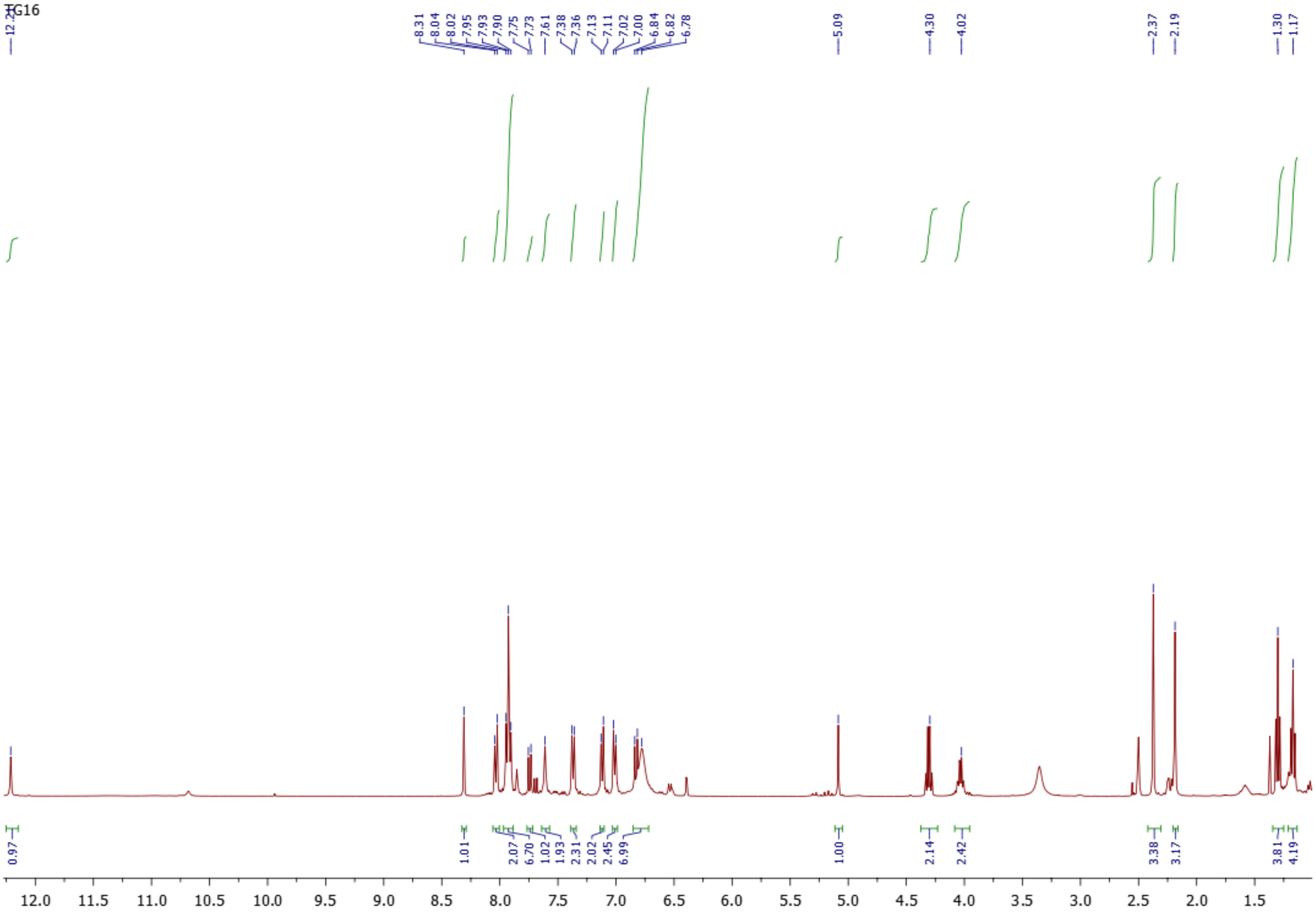

Figure S13: ^{1}H NMR spectra of azo sulfa chromene ester **11c**.

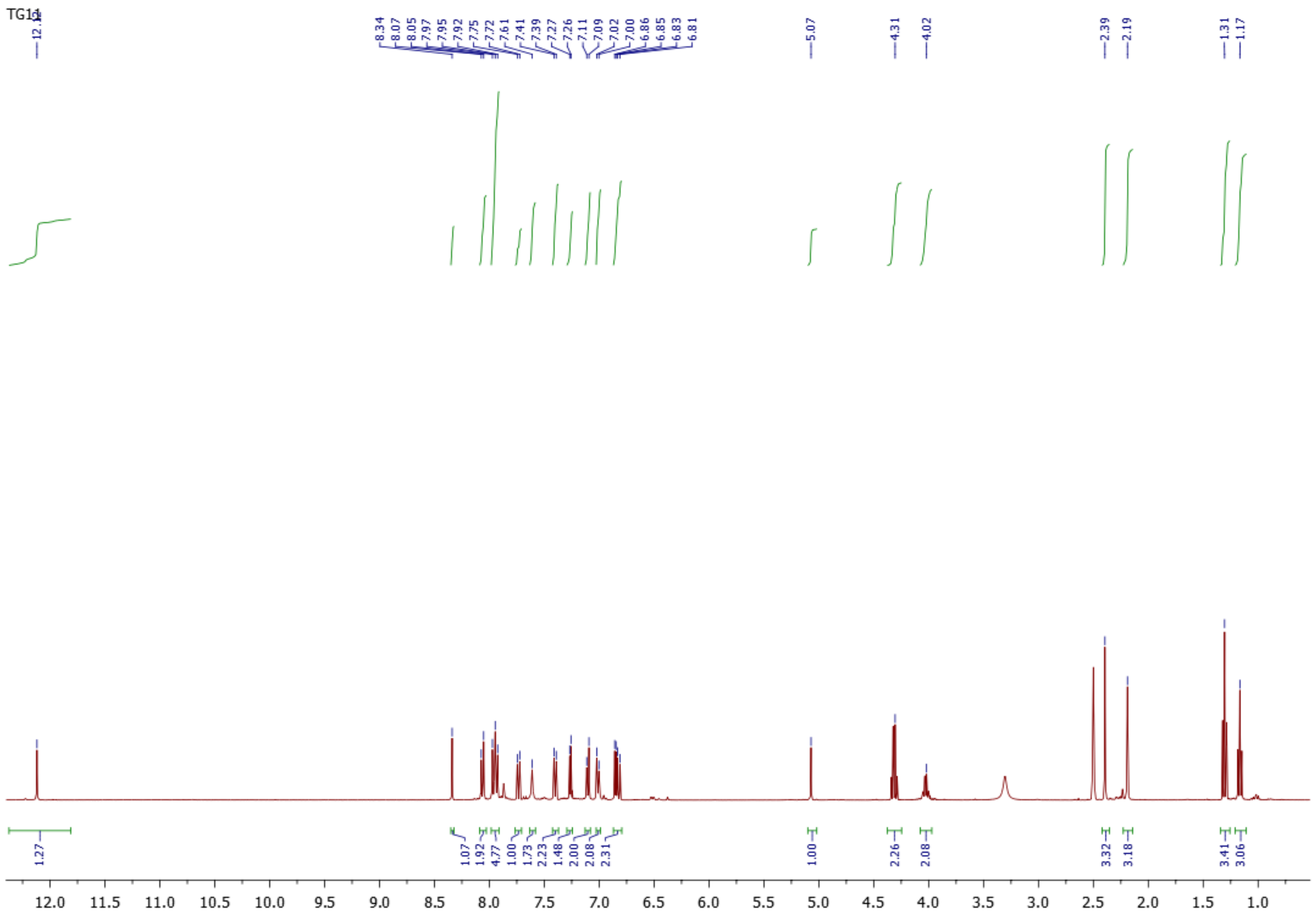

Figure S14: ^{1}H NMR spectra of azo sulfa chromene ester **11d**.

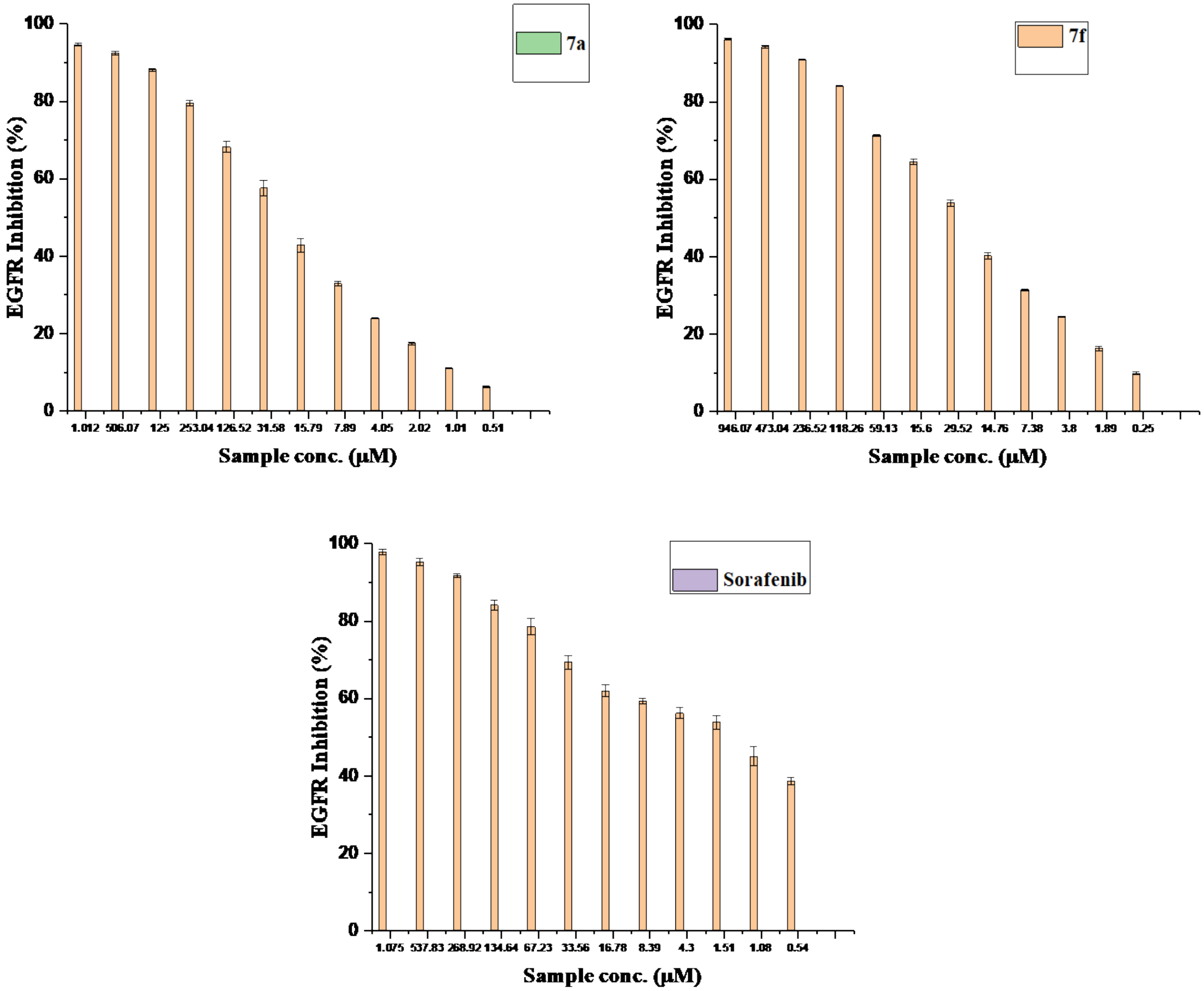

Figure S15: The inhibitory activity of selected compounds on EGFR.

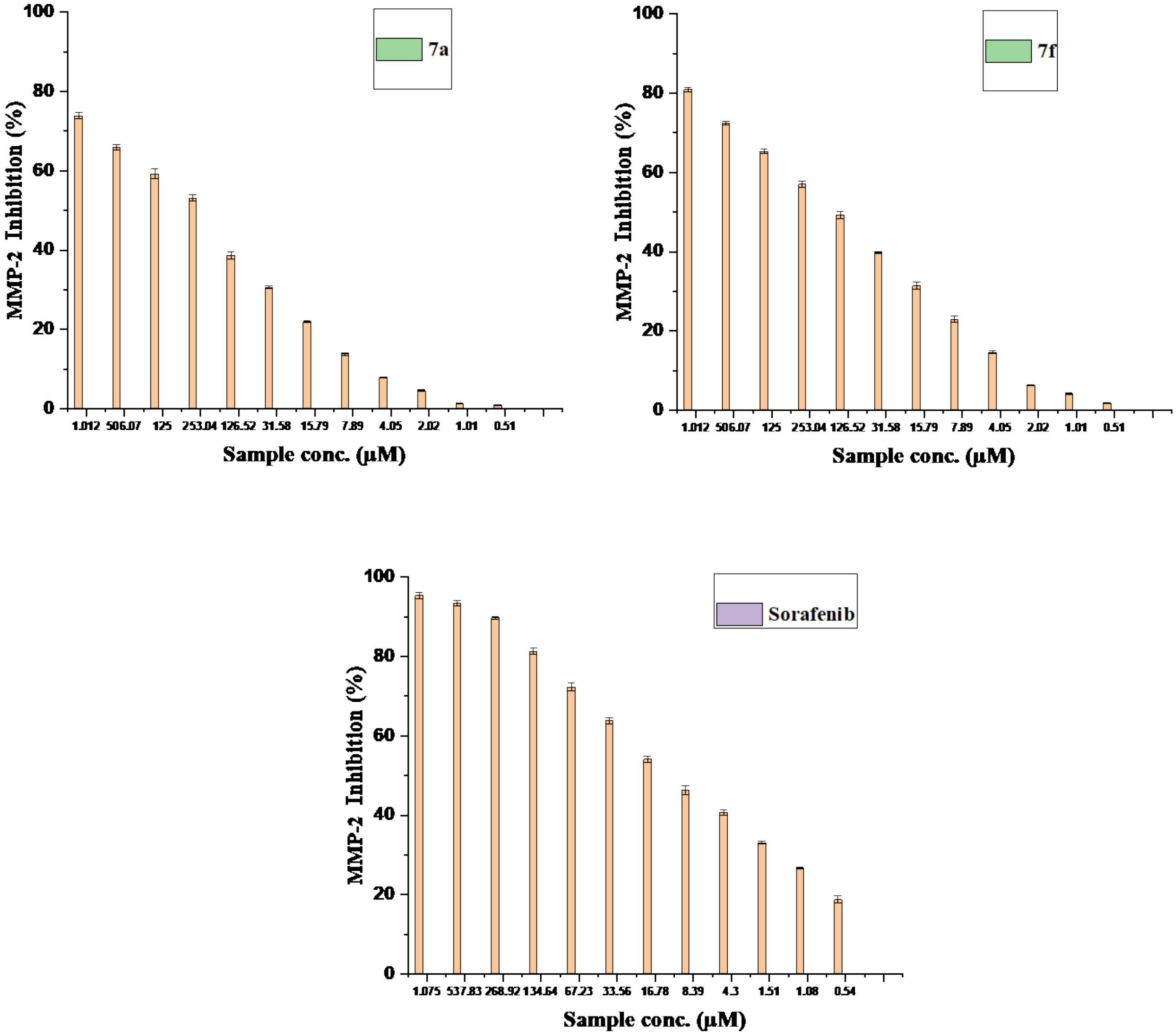

Figure S16: The inhibitory activity of selected compounds MMP-2.

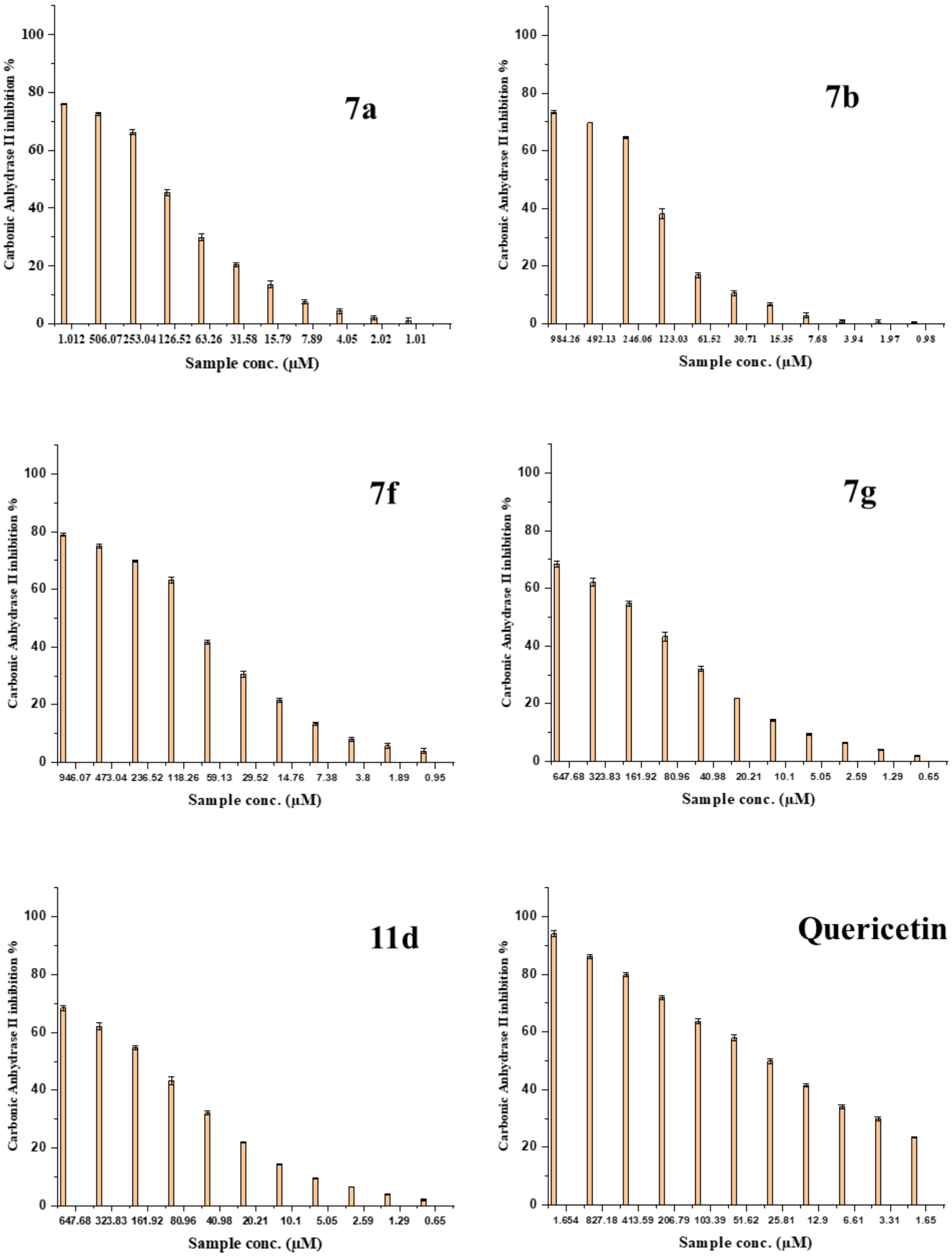

Figure S17: The inhibitory activity of selected compounds on carbonic anhydrase II.

**2a**	**2b**	**2c**
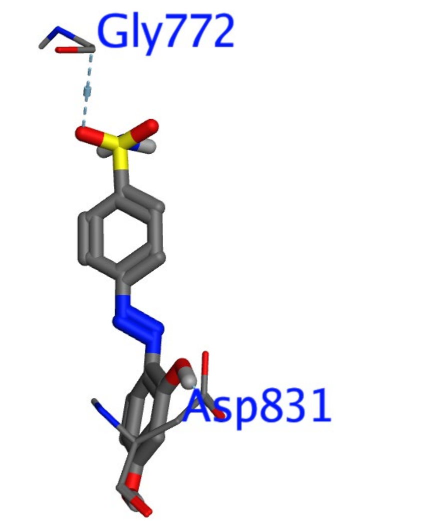	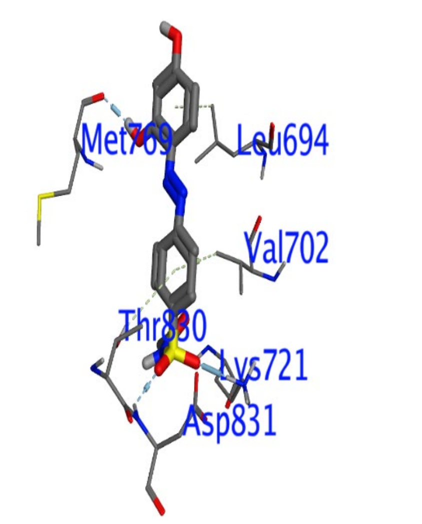	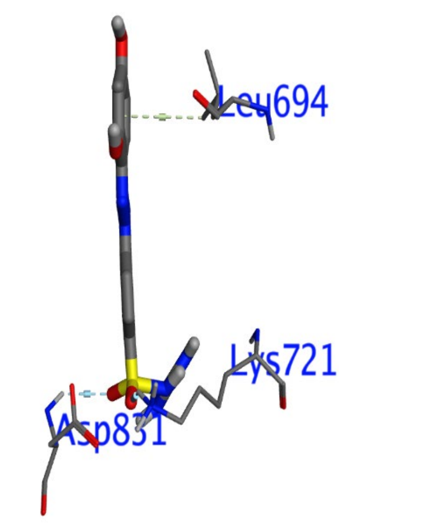
**2d**	**2e**	**7a**
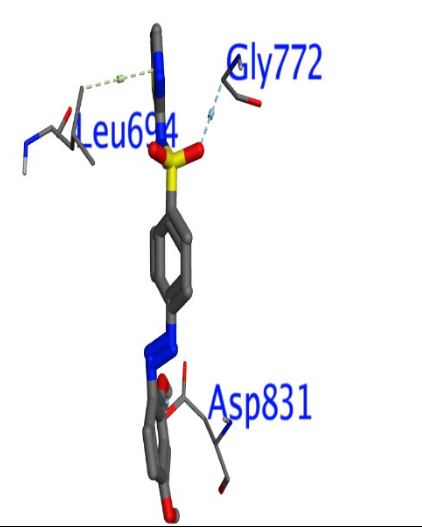	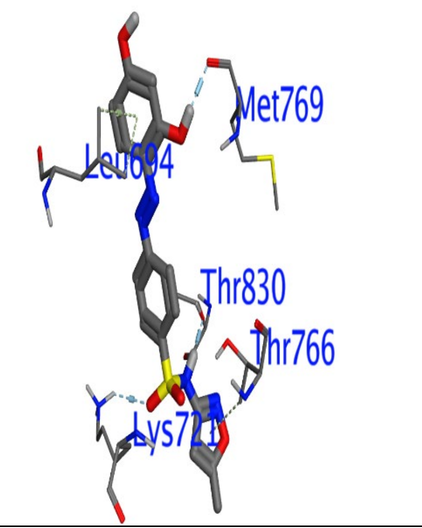	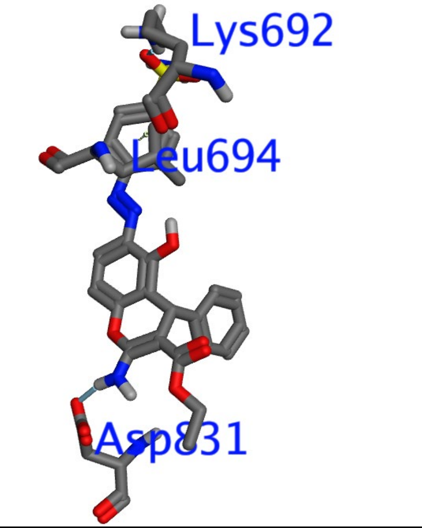
**7b**	**7c**	**7d**
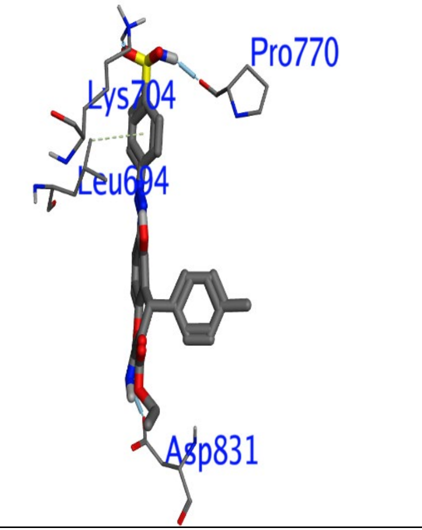	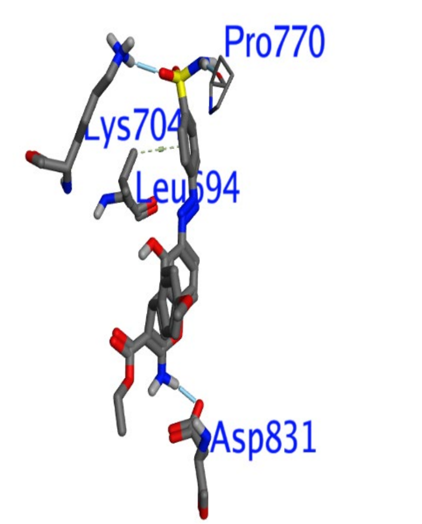	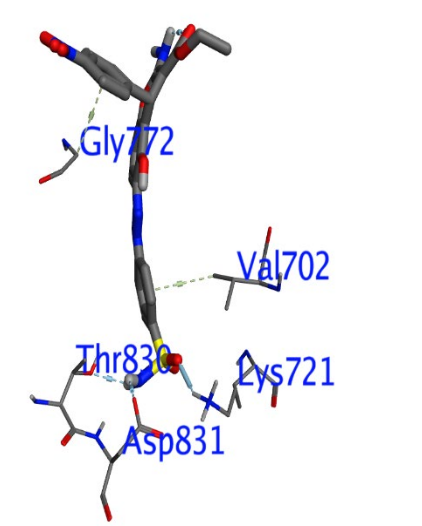
**7e**	**8**	**11a**

<table>
<tr><td>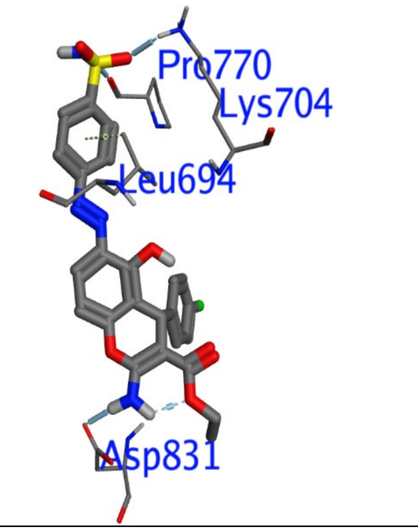
</td><td>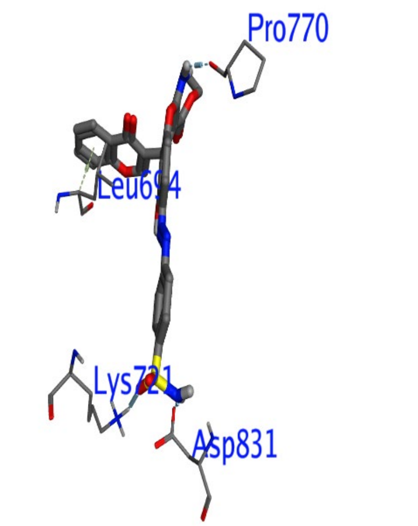
</td><td>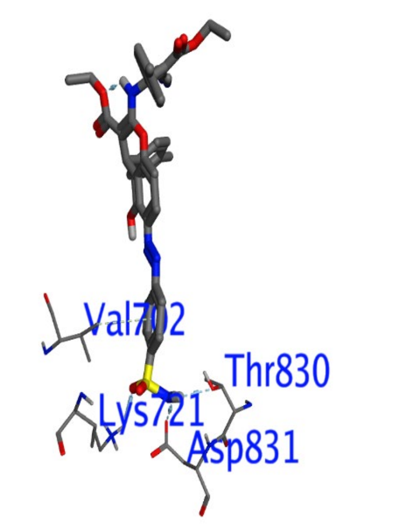
</td></tr>
<tr><td>11b</td><td>11c</td><td>11d</td></tr>
<tr><td>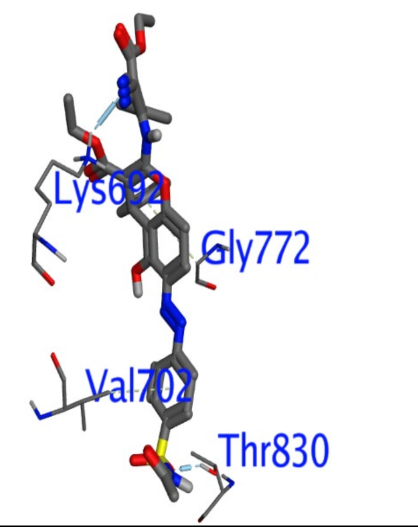
</td><td>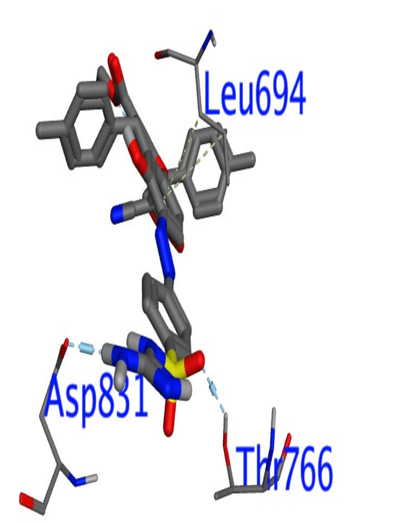
</td><td>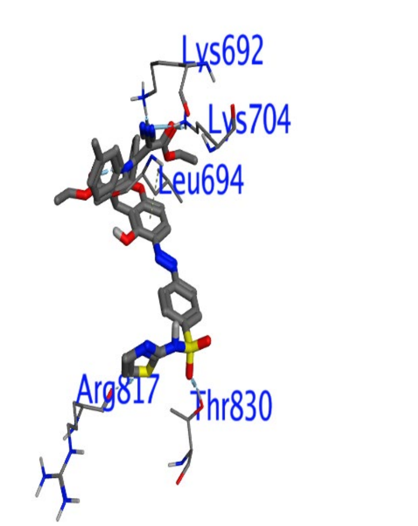
</td></tr>
<tr><td>11e</td><td></td><td></td></tr>
<tr><td>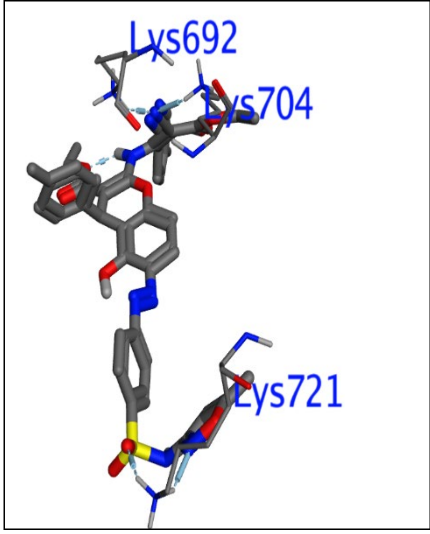
</td><td></td><td></td></tr>
</table>

Figure S18: Docking poses of compounds **2a-e,7a-g, 8** and **11a-e**

Table S1: The binding-affinity for compounds **2a-e** with docking score (kcal/mol) against EGFR

**No.**	***ΔG***	**RMSD**	**H. B**	**EInt.**	**Eele**	**LE**
**2a**	-6.420	1.095	-15.427	-15.208	-11.841	2.471
**2b**	-7.068	1.025	-115.861	-18.606	-12.206	1.980
**2c**	-7.187	1.714	-228.808	-16.767	-12.632	1.540
**2d**	-7.891	1.200	-55.102	-16.691	-11.529	2.158
**2e**	-7.884	1.603	-42.333	-18.561	-13.346	1.733
**Erlotinib**	**-5.361**	**1.342**	**-8.175**	**-17.976**	**-9.201**	**2.885**

Mass Spectra of compounds 11a-e: (11a = G12, 11b = G15, 11c = G16, 11d = G11, 11e = G14)

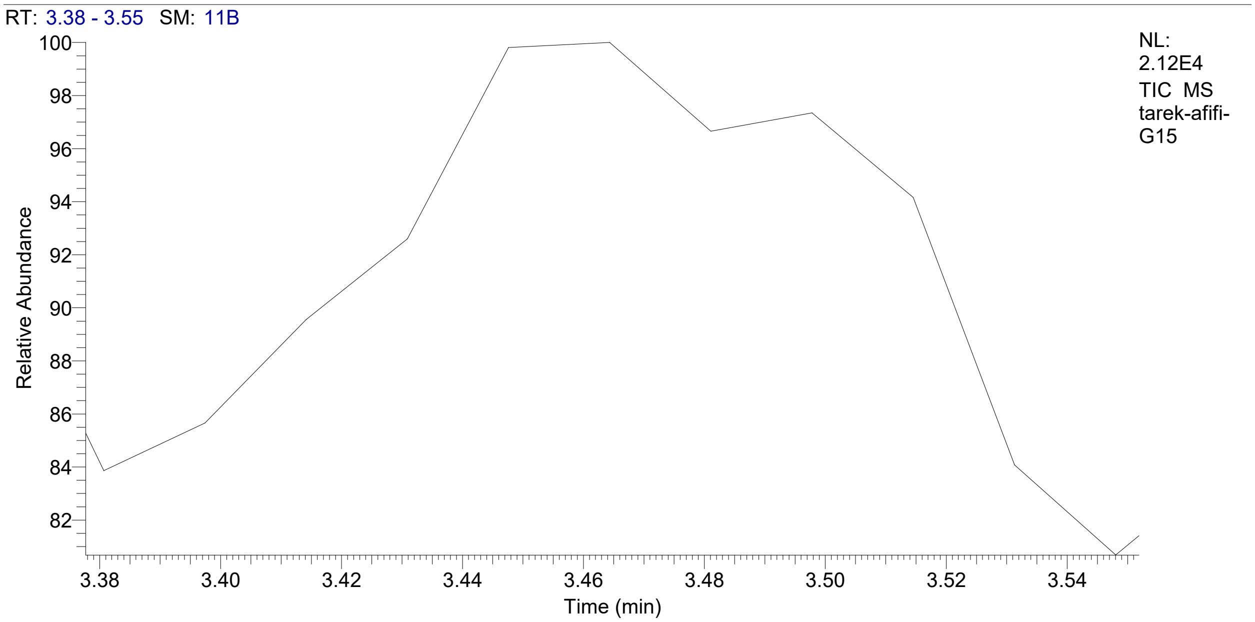

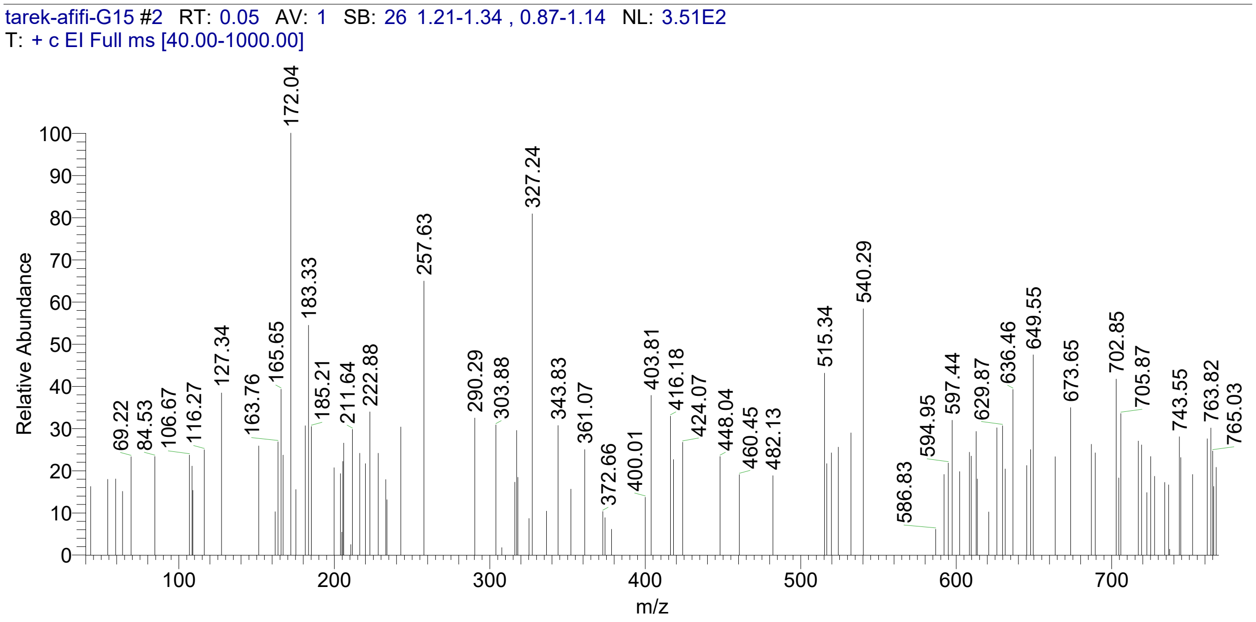

RT: 1.48 - 1.65 SM: 11B

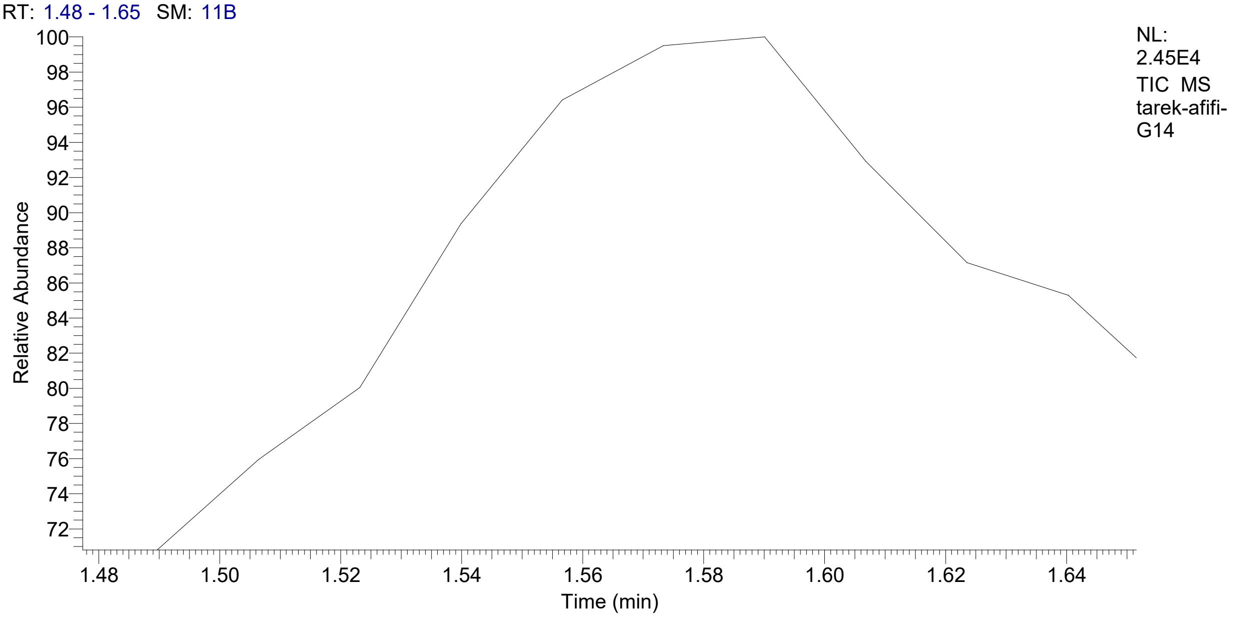

tarek-afifi-G14 #71-72 RT: 1.21-1.22 AV: 2 SB: 26 1.21-1.34 , 0.87-1.14 NL: 1.17E2
T: + c EI Full ms [40.00-1000.00]

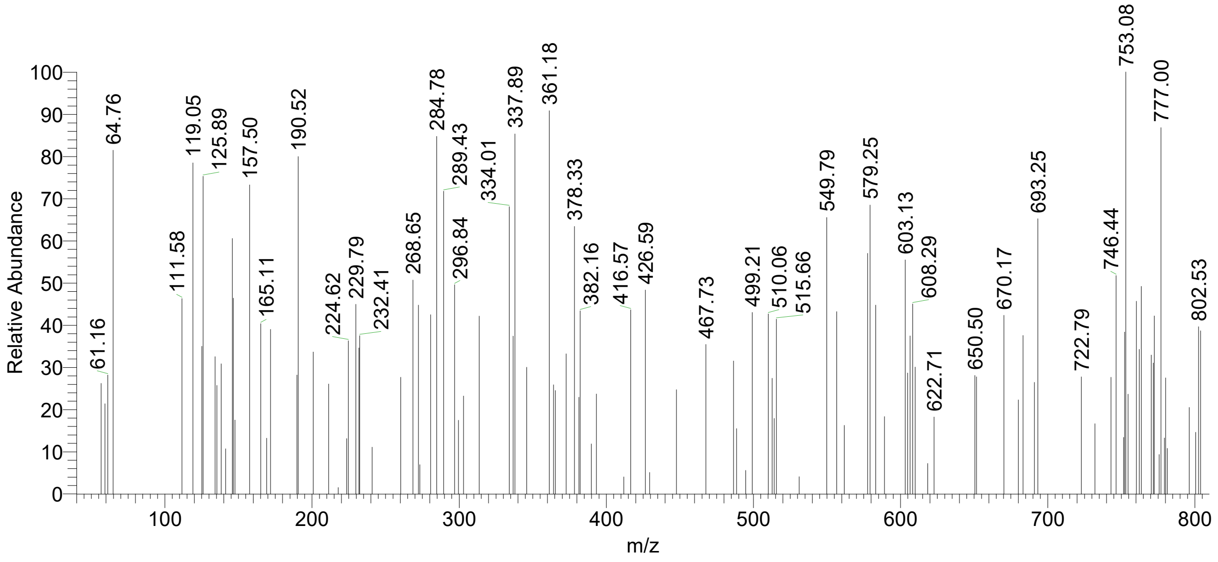

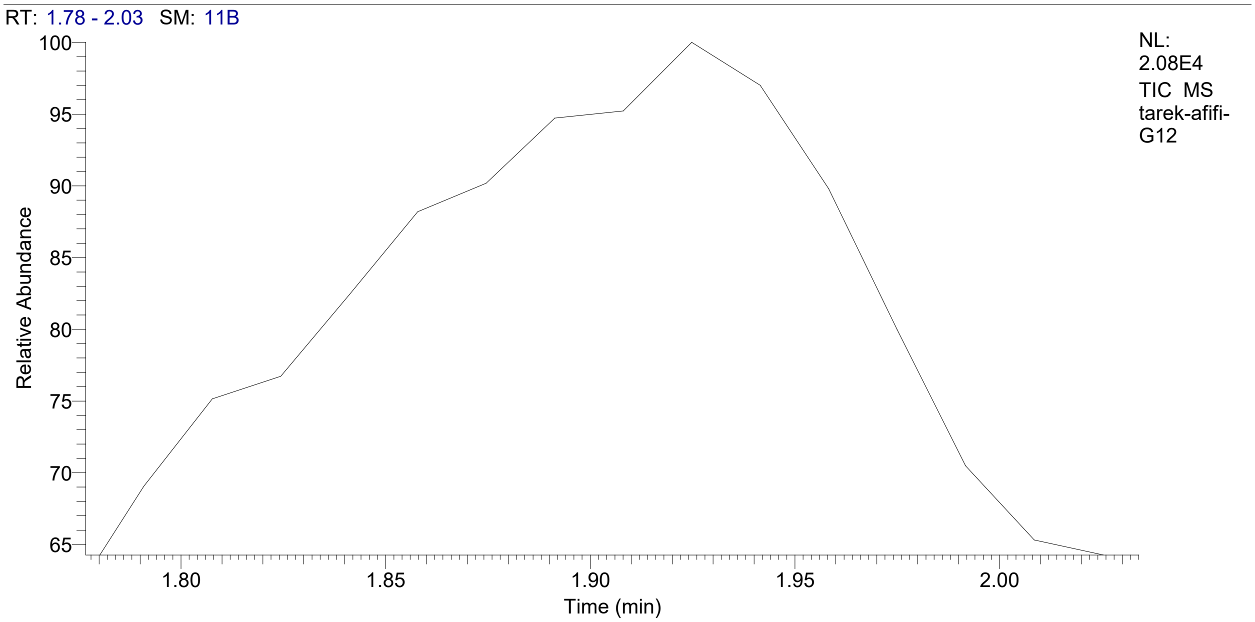
RT: 1.78 - 2.03 SM: 11B
NL: 2.08E4
TIC MS
tarek-afifi-G12
Relative Abundance
Time (min)

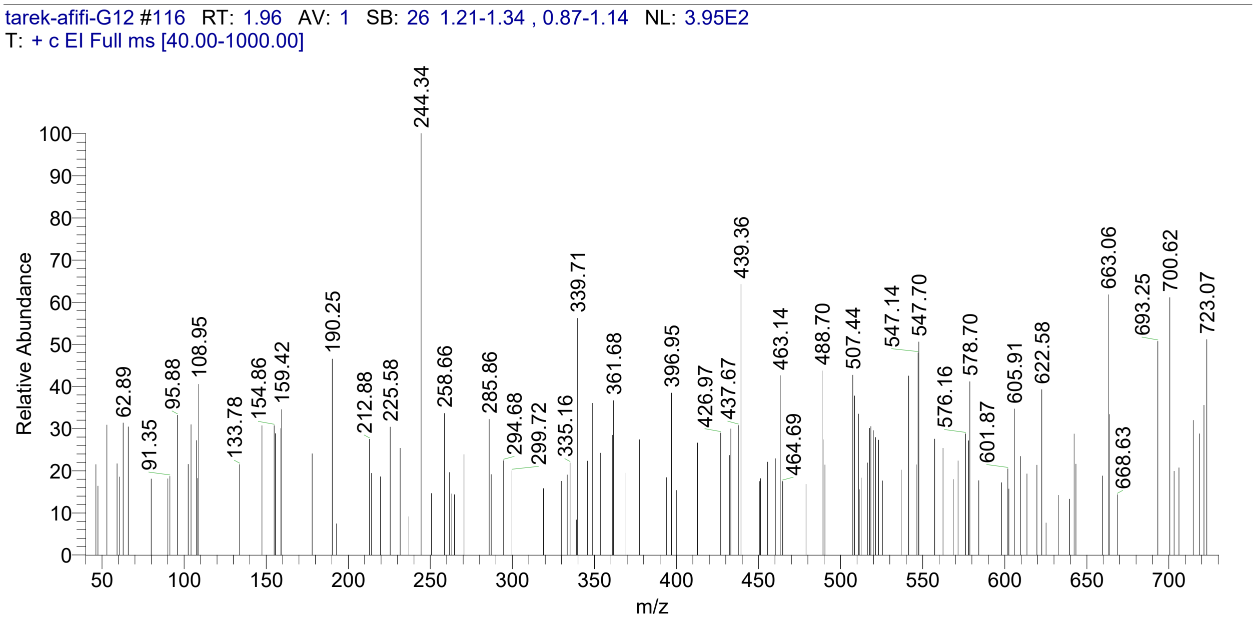
tarek-afifi-G12 #116 RT: 1.96 AV: 1 SB: 26 1.21-1.34 , 0.87-1.14 NL: 3.95E2
T: + c EI Full ms [40.00-1000.00]
244.34
439.36
663.06
700.62
693.25
723.07
339.71
547.70
547.14
Relative Abundance
m/z

RT: 0.00 - 4.62 SM: 11B

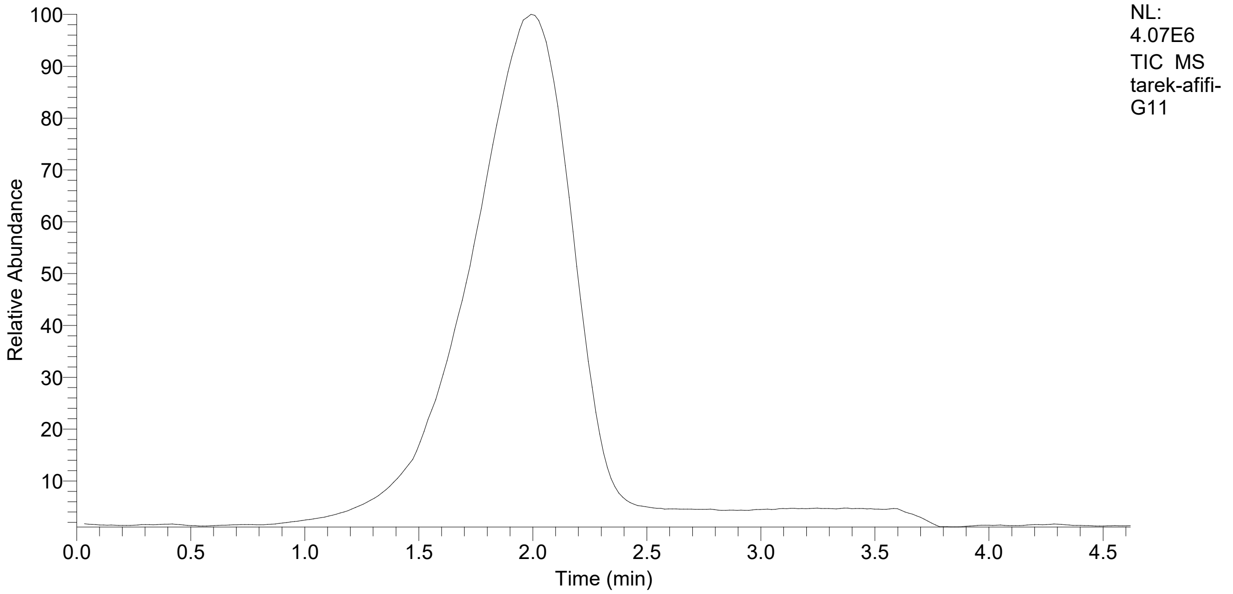

tarek-afifi-G11 #249 RT: 4.18 AV: 1 SB: 26 1.21-1.34 , 0.87-1.14 NL: 9.49E2
T: + c EI Full ms [40.00-1000.00]

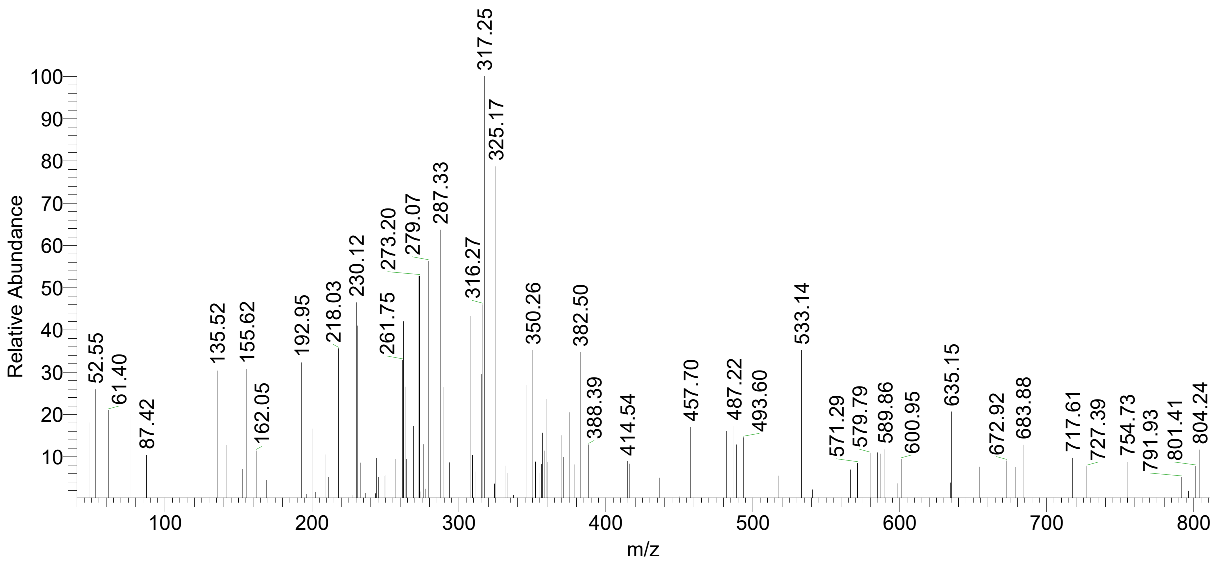

RT: 2.19 - 3.76 SM: 11B

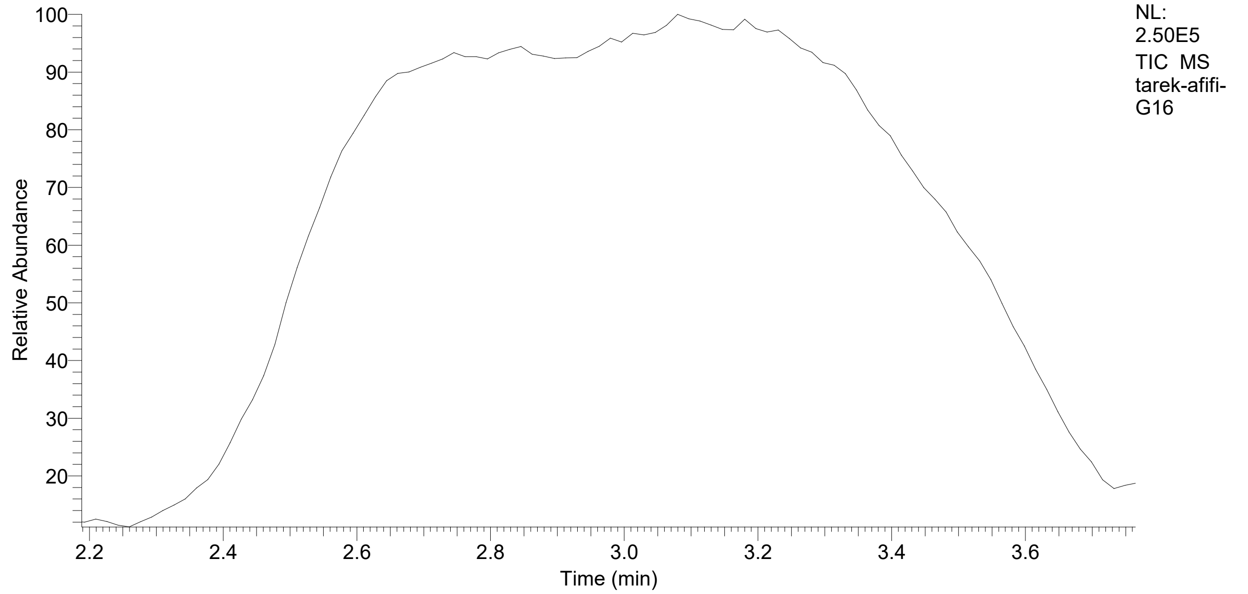

tarek-afifi-G16 #240 RT: 4.03 AV: 1 SB: 26 1.21-1.34 , 0.87-1.14 NL: 1.08E3
T: + c EI Full ms [40.00-1000.00]

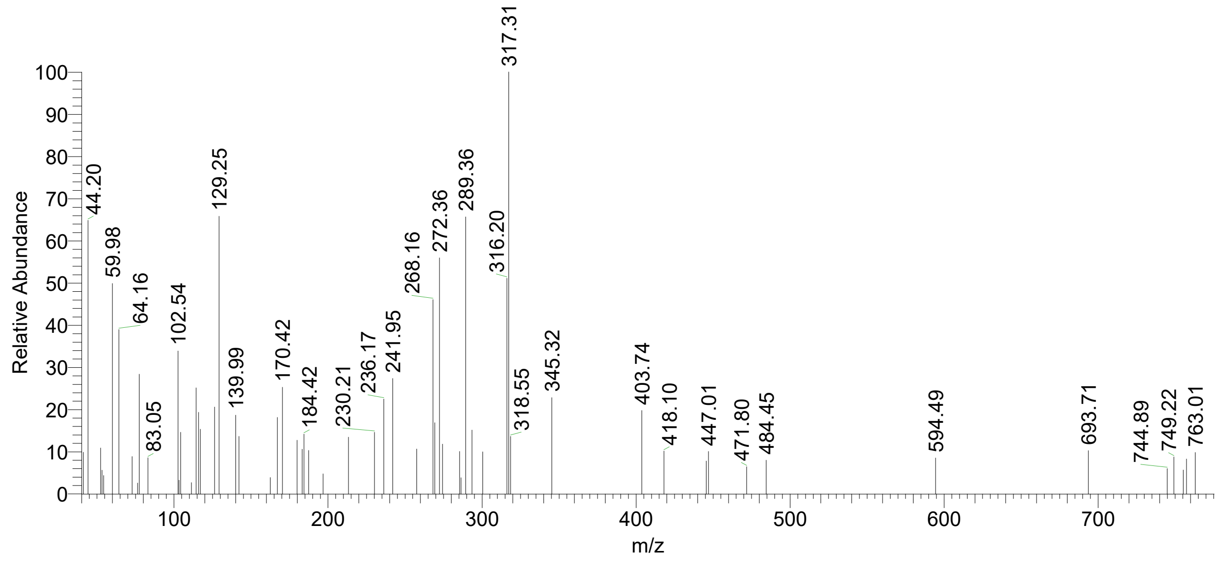

Supplement: Supplementary file 1 [file ijms-24-16716-s001.zip › ijms-2699813-supplementary.pdf]
